# A novel non-invasive EEG-SSVEP diagnostic tool for color vision deficiency in individuals with locked-in syndrome

**DOI:** 10.3389/fbioe.2024.1498401

**Published:** 2025-01-07

**Authors:** Ghada N. AlEssa, Saleh I. Alzahrani

**Affiliations:** Biomedical Engineering Department, College of Engineering, Imam Abdulrahman Bin Faisal University, Dammam, Saudi Arabia

**Keywords:** color vision deficiency, diagnosing, EEG, SSVEP, signal processing, feature extraction, classification

## Abstract

**Introduction:**

Color vision deficiency (CVD), a common visual impairment, affects individuals’ ability to differentiate between various colors due to malfunctioning or absent color photoreceptors in the retina. Currently available diagnostic tests require a behavioral response, rendering them unsuitable for individuals with limited physical and communication abilities, such as those with locked-in syndrome. This study introduces a novel, non-invasive method that employs brain signals, specifically Steady-State Visually Evoked Potentials (SSVEPs), along with Ishihara plates to diagnose CVD. This method aims to provide an alternative diagnostic tool that addresses the limitations of current tests.

**Methods:**

Electroencephalography (EEG) recordings were obtained from 16 subjects, including 5 with CVD (specifically Deuteranomaly), using channels O1, O2, Pz, and Cz. The subjects were exposed to visual stimuli at frequencies of 15 Hz and 18 Hz to assess the proposed method. The subjects focused on specific visual stimuli in response to questions related to the Ishihara plates. Their responses were analyzed to determine the presence of CVD. Feature extraction was performed using Power Spectral Density (PSD), Canonical Correlation Analysis (CCA), and a combined PSD + CCA, followed by classification to categorize subjects into two classes: normal vision and CVD.

**Results:**

The results indicate that the proposed method effectively diagnoses CVD in individuals with limited communication abilities. The classification accuracy of SSVEP exceeded 75% across the three classifiers: Decision Tree (DT), K-Nearest Neighbors (KNN), and Support Vector Machine (SVM). The SVM classifier demonstrated higher accuracy compared to the other classifiers, exceeding 90%.

**Discussion:**

These observations suggest that the SVM classifier, utilizing the combined feature set of PSD + CCA, may be the most effective in this classification task. These findings demonstrate that the proposed method is an accurate and reliable diagnostic tool for CVD, particularly for individuals unable to communicate.

## 1 Introduction

Color vision is based on the fact that the human retina contains three different cone photoreceptors, which absorb photons (Celesia and Celesia, 2005). Having an abnormality or lacking one or more of these cones leads to color vision deficiency (CVD). CVD is basically defined as the inability for a person to distinguish colors in normal lighting (Woldeamanuel and Geta, 2018), causing struggles in affected individuals in their daily lives, such as driving, working, or consciousness levels. In the retina, each cone photoreceptor is sensitive to a specific region of the visible spectrum, covering a range of wavelengths associated with specific color hues. There are three main types of cones: the S-cone, which detects short wavelengths; the M-cone, which detects medium wavelengths; and the L-cone, which detects long wavelengths (Thoreson and Dacey, 2019). The ranges of the cones in the visible spectrum overlap, requiring complex computations by the brain to accurately recognize the correct hue. This shows that brain signals can provide significant insights into color perception, which involves several steps. It is initiated by cones detecting the wavelength, after which the brain decodes the signal by comparing the overlapping ranges of different cones, allowing us to perceive the correct color (Thoreson and Dacey, 2019; Conway, 2009; Werner, 2014). CVD is associated with abnormalities in cone sensitivity or the lack of specific cones (Chan et al., 2014). It is one of the most common vision disorders, affecting 1 in 12 males and 1 in 200 females (Alamoudi et al., 2021). The prevalence rate of CVD is not constant among different populations, with rates ranging from 1.40% to 13.93%, depending on various factors, including genetic and environmental influences (Male et al., 2023; Fareed et al., 2015; Fakorede et al., 2022; Shah et al., 2013). When a specific cone fails to detect a wavelength, it results in the failure to transmit the signal to the brain, impacting the decoding process and ultimately leading to a failure in color perception (Alamoudi et al., 2021). Genetic CVDs are classified into two main types based on the affected cone. The types of CVD are anomalous trichromacy and dichromacy. Anomalous trichromacy is subdivided into protanomaly, associated with an abnormal L-cone; deuteranomaly, associated with an abnormal M-cone; and tritanomaly, associated with an abnormal S-cone. Dichromacy is subdivided into protanopia, associated with a missing L-cone; deuteranopia, associated with a missing M-cone; and tritanopia, associated with a missing S-cone (Chan et al., 2014; Dohvoma et al., 2018).

People with CVD suffer in various aspects of their lives, especially when they do not recognize the disorder early on. Studies show that individuals with CVD often lack awareness of their condition in early childhood compared to their peers, leading to missed opportunities and disadvantages if they are unaware of it. This lack of awareness can impact their life paths and daily activities (Woldeamanuel and Geta, 2018; Chan et al., 2014; emhj, 2021). However, detecting the disorder early can provide them with an opportunity to adapt and manage the condition naturally without significantly affecting their lives (Alamoudi et al., 2021; Gordon, 1998). The most common methods for diagnosing CVD include the Ishihara test, the Farnsworth–Munsell (FM) 100-hue test, and the anomaloscope (Fanlo Zarazaga et al., 2019; Pandey et al., 2015). Nevertheless, other methods have been developed and tested, such as genetic testing, which depends on identifying any changes in genes associated with CVD (Davidoff et al., 2016), or color arrangement tests, such as the Farnsworth D-15, which depend on arranging colors in a specific order (Károly, 2024), or using imaging techniques such as Spectral Domain Optical Coherence Tomography (SD-OCT) to assess the sensitivity of the retina’s photoreceptors (Gupta et al., 2011).

These methods are not always accurate and may not be suitable for individuals with movement or communication disabilities or young children, as they require behavioral responses. Additionally, some tests, such as the Ishihara test, provide only qualitative assessments, while others, such as the FM test and D-15 test, rely on subjective responses that can lead to errors. Some tests are challenging and require training, such as the anomaloscope. Moreover, genetic testing has limitations as it may miss some undiscovered mutations leading to ambiguity in diagnosis and is considered complex, making it impractical in routine clinical diagnosis. Finally, imaging techniques like SD-OCT provide structural but not functional information, making it inaccurate to diagnose vision diseases, such as CVD. Therefore, it is crucial to find alternative methods that can overcome the limitations of current assessments, allowing for accurate and precise diagnostic procedures to help those affected by CVD (Fanlo Zarazaga et al., 2019; Pandey et al., 2015; Davidoff et al., 2016; Gupta et al., 2011; Salvia and Ysseldyke, 1971). In recent times, Electroencephalography (EEG) has emerged as a promising avenue for diagnosing CVD. By measuring brain activity, EEG provides a unique window into neural responses triggered by visual stimuli, potentially enhancing the accuracy and depth of diagnosis in this particular field, paving the way for a more comprehensive understanding of CVD and improving the lives of those affected.

Building on this understanding, the brain signals measured by EEG can provide significant insights into retina color perception, enabling the development of an objective and accurate method for diagnosing CVD (Teixeira and Gomes, 2023). Specifically, the steady-state visual evoked potential (SSVEP) examines the responses of neurons when exposed to repetitive visual stimulation at specific frequencies. SSVEP is an effective choice for detecting EEG variations in response to different colors due to its high signal-to-noise ratio (SNR) and low susceptibility to artifacts (Cao et al., 2012; Albahri et al., 2023; Chu et al., 2017). However, the use of SSVEP for diagnosing CVD has been minimally explored, despite its potential to improve diagnostic methods and benefit many individuals worldwide. Most studies have focused on the variations that colors could induce in SSVEP response, but they are unaware of its potential for diagnostic purposes. Additionally, some limitations associated with using SSVEP as a diagnostic technique for CVD could include the challenge of identifying procedures that are not overly complex and time-consuming. Therefore, this study aims to present a direct and objective method using SSVEP to facilitate the easy diagnosis of CVD, focusing on the most common type of CVD, red-green CVD, which includes protanomaly, deuteranomaly, protanopia, and deuteranopia. The proposed method aims to overcome the limitations of current assessments and enhance the lives of people with CVD.

This paper is structured into multiple sections. It begins with a review of related work to contextualize previous studies in the field. Following this, the methodology section explains the approach adopted to fulfill the study’s objectives. Subsequently, the results section presents and thoroughly analyzes the experimental findings obtained through the methodology explained. Finally, the discussion section delves deeper into the implications of the results, emphasizing the significance and efficacy of the proposed method. The paper concludes with a succinct summary and outlines potential future research endeavors.

## 2 Related works

In EEG techniques, SSVEP has been recognized as a viable option for diagnosing CVD in several studies, while alternative methods like ERP have also been employed in this field. However, ERP has rarely been utilized for diagnostic purposes in this area; instead, it has explored the relationship between brain function and color recognition. The scarce studies that have applied this for diagnostic purposes are only referenced in Bieber et al. (1997) and Thomas et al. (2017). In Bieber et al. (1997), they utilized the silent distribution method, which involves adjusting the levels of various light wavelengths to stimulate the specific cone type required. Their study aimed to diagnose infants by comparing their results to those of adults, which could be a limitation due to variations in human retinas with age (Hendrickson et al., 2008; Vinekar et al., 2015). In Thomas et al. (2017), they developed a device in which they used color stimuli consisting of the primary colors (red, green, and blue) during EEG recordings. Subsequently, they calculated the energy variance, observing a higher deviation in the particular color associated with CVD in the individual. However, they tested their method on only two subjects, making it challenging to generalize the results, which implies the need for further testing to ensure more dependable outcomes (AlEssa and Alzahrani, 2024).

The infrequent use of SSVEP for diagnostic purposes parallels that of ERP. Prior to 2020, there were relatively few published studies on the application of SSVEP in diagnosing CVD, despite the longstanding recognition of the influence of different colors on SSVEP responses (Thomas and Umamaheswari, 2016; Roy et al., 2021; Göksel Duru and Alobaidi, 2021). The studies utilizing SSVEP for diagnostic aims are mainly (Zheng et al., 2021), and (Norton et al., 2021). In Zheng et al. (2021), they implemented a technique based on sweep SSVEP to diagnose CVD by designing a stimulus pattern involving a red-green checkerboard with varying luminance ratios to evoke the SSVEPs and make conclusions by using an equiluminance turning curve. Because their research incorporated sweep SSVEP, encompassing a wide frequency spectrum for stimulation, this introduced some limitations associated with complexity and time-consuming. However, in Norton et al. (2021), they studied the detection of CVD through SSVEP, not sweep SSVEP, utilizing an innovative approach to spot metamers. They discovered that individuals with normal color vision exhibit an SSVEP close to zero, whereas those with CVD do not. However, their study includes more than one experiment for each subject and a total time reaching approximately one hour, which could be considered a limitation and challenging to use this method in routine clinical diagnosis.

The novel technique proposed in this paper for diagnosing CVD through SSVEP aims to address the limitations identified in previous studies. Unlike the challenges associated with the complexity and time-consuming nature of previous studies, the method introduced here focuses on utilizing SSVEP in a streamlined, efficient, and time-saving manner compared to all studies that utilized EEG for diagnostic purposes. By specifically using SSVEP instead of sweep SSVEP, this technique offers a more direct and precise approach to CVD diagnosis. Moreover, by optimizing the experimental design to minimize the number of experiments required for each subject and reducing the total assessment time to 30 min, this method aims to enhance the feasibility and practicality of using SSVEP for routine clinical diagnosis. By overcoming these limitations through a more focused and efficient application of SSVEP, this novel technique has the potential to provide a more accessible and reliable method for diagnosing CVD, offering significant advancements in this field.

## 3 Methodology

This section outlines the methodology employed to achieve the objectives of the study. It provides a detailed description of the research design, data collection procedures, analytical techniques, and other pertinent aspects of the methodology, building on relevant literature to ensure a robust approach for addressing the research questions.

### 3.1 Participants

The study protocol was approved by the Institutional Review Board of Imam Abdulrahman bin Faisal University. All participants received comprehensive information about the study and voluntarily signed a written consent form in accordance with the approved protocol. Written informed consent was obtained from the individuals for the publication of any potentially identifiable images or data included in this article. The study included 16 participants (10 males and 6 females) with an age of 30.5 ± 9.7. Of these, 5 (all male) had red-green color vision deficiency, while the remaining 11 had normal color vision. All subjects had no history of neurological or ophthalmological diseases. Table 1 summarizes the demographic details of the study participants, including age, gender, and health condition (CVD or healthy).

**TABLE 1:** Demographic information of study participants.

Subject ID	Gender	Age (years)	Health condition	Subject ID	Gender	Age (years)	Health condition
1	M	35	Healthy	9	M	41	CVD
2	F	25	Healthy	10	F	25	Healthy
3	F	26	Healthy	11	M	37	Healthy
4	M	18	Healthy	12	F	23	Healthy
5	F	23	Healthy	13	F	30	Healthy
6	M	36	CVD	14	M	22	CVD
7	M	57	Healthy	15	M	40	CVD
8	M	28	Healthy	16	M	22	CVD

### 3.2 EEG recordings

**FIGURE 1:**
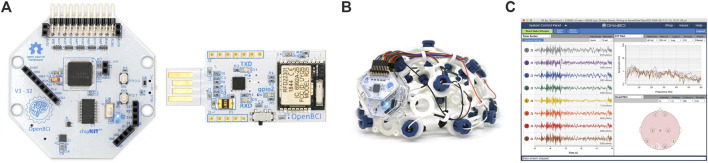
**(A)** The OpenBCI Cyton Board and USB dongle used in the experiment, **(B)** the EEG electrode cap with the 10–20 electrode placement system, **(C)** the OpenBCI GUI (OpenBCI, 2023).

**FIGURE 2:**
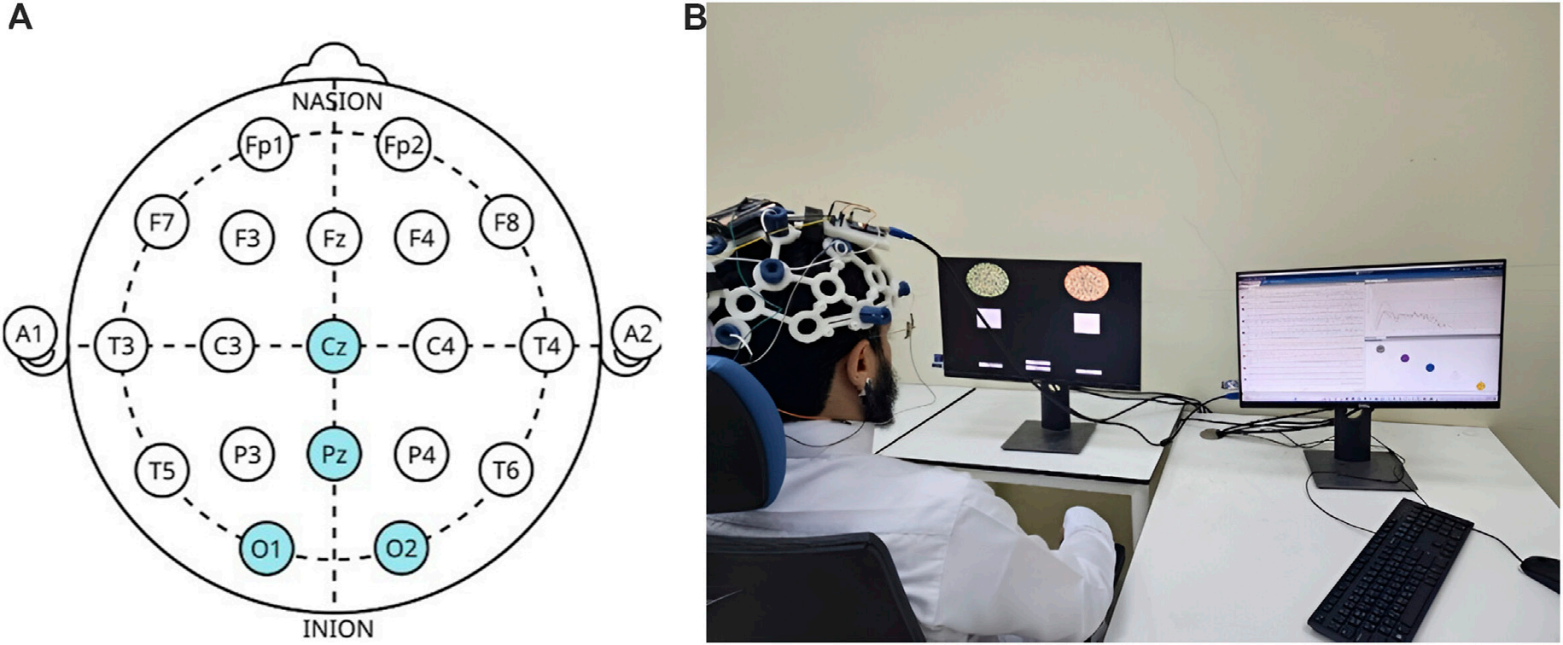
**(A)** Electrode placement’s locations according to the 10–20 system, with highlighted regions indicating the specific sites used in this study. **(B)** Experimental setup with a subject undergoing the EEG recording. The left screen displays the visual stimuli generated using Python, while the right screen shows real-time data acquisition in the OpenBCI GUI.

To record the EEG, an OpenBCI Cyton Biosensing Board (OpenBCI, Brooklyn, NY, United States), as shown in Figure 1A, was used. The board was connected to a PC via a USB dongle. The board has 8 channels and a 32-bit processor, and all the data were sampled at 250 Hz. The EEG was recorded using a cap with dry comb electrodes, shown in Figure 2B, which were placed according to the international 10–20 electrode system. These electrodes were chosen for their compatibility with the OpenBCI board, as well as their ease of use. Four channels were used: O1, O2, Cz, and Pz/, as shown in Figure 2A. Selecting these specific regions due to their significant association with cortical vision in the brain (Norcia et al., 2015; Srinivasan et al., 2006). The reference and ground electrodes were placed on the right and left earlobes, respectively, using conductive gel to enhance conductivity and reduce impedance. The OpenBCI GUI, shown in Figure 1C, was used for data acquisition. The experiment interface was designed in Python (version 3.12.0), and data analysis was conducted using both Python and MATLAB (version R2023b; The MathWorks, Inc., Natick, MA, United States). Figure 2B shows the experimental set-up, with the subject wearing the EEG cap equipped with dry comb electrodes. The subject is exposed to a screen with the stimulation function, while another screen displays the measured data during the experiment.

### 3.3 Experimental procedure for eliciting SSVEPs

**FIGURE 3:**
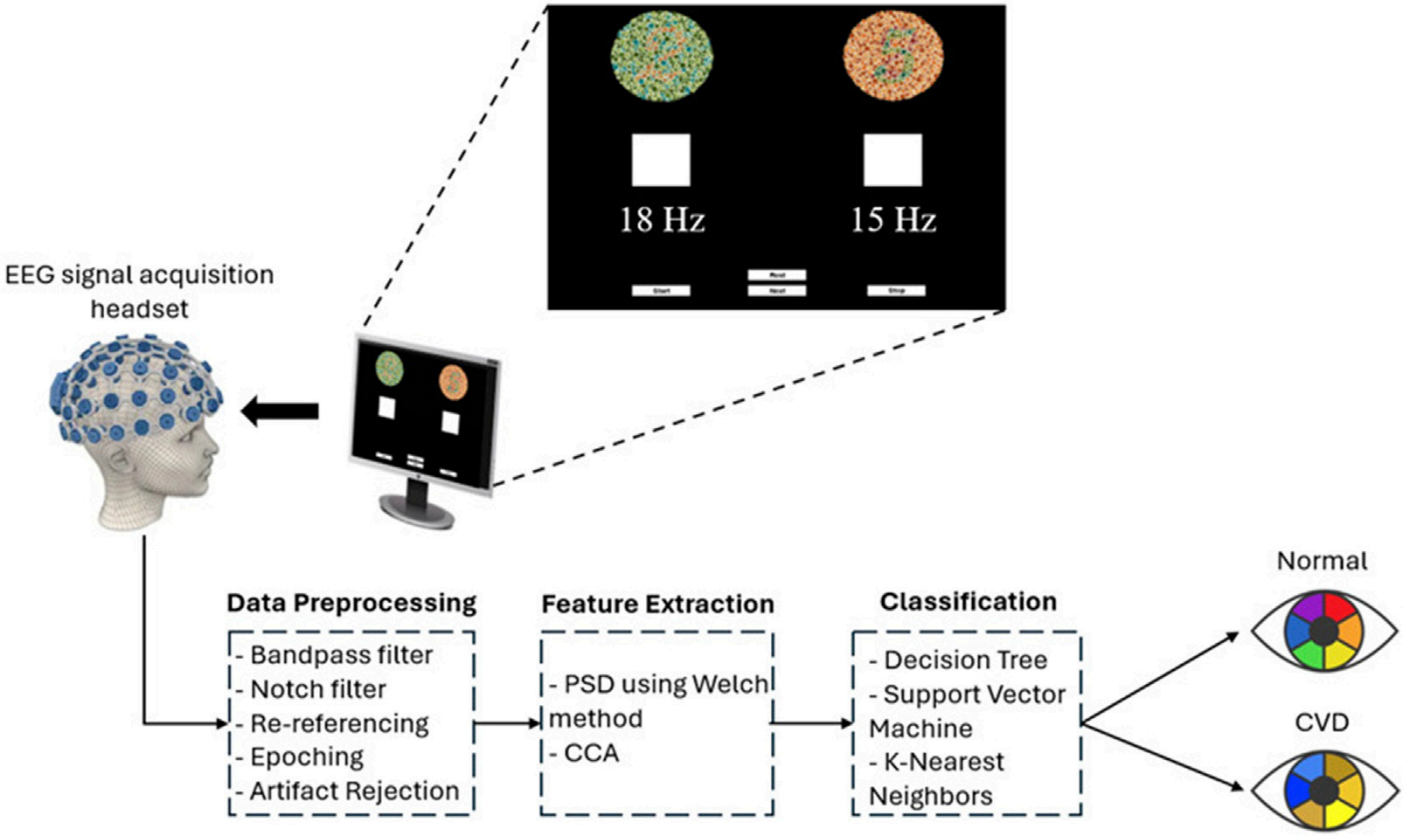
Overview of the SSVEP-based BCI system used in this study for diagnosing color vision deficiency (CVD). EEG signals were acquired using the OpenBCI headset, with visual stimulation presented at flickering frequencies of 15 Hz and 18 Hz on a screen. The acquired EEG signals were filtered and segmented. Subsequently, power spectral density (PSD) was extracted using the Welch method for feature extraction. Additionally, Canonical Correlation Analysis (CCA) was employed as another feature extraction technique. Finally, classification was performed using three classifiers to differentiate between normal and CVD conditions.

**FIGURE 4:**
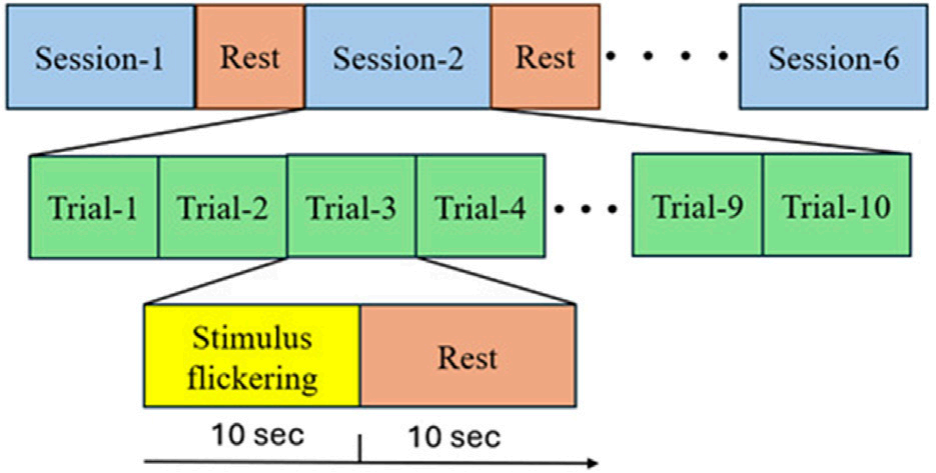
Experimental Steps Followed by Subjects in Each Session: Firstly, the subjects were asked to choose between two Ishihara plates and focus on the flickering square corresponding to their choice for ten seconds. They then took a ten-second rest. This process was repeated ten times (trial and rest) before proceeding to the next session, which involved different Ishihara plates and different questions.

A specially designed stimulation setup, incorporating a software interface, was used to elicit SSVEPs. The interface, as shown in Figure 3 consists of two squares flickering at different frequencies (15 Hz and 18 Hz) with a series of Ishihara plates positioned above each square. The EEG was recorded for the subjects during the task. Each subject was asked to sit in front of a display in a laboratory with normal lighting. Each subject then focused on a flickering square (15 Hz or 18 Hz) positioned below a pre-identified number to assess whether they selected the correct plate, which indicated if the subject had CVD. Each subject underwent six sessions, with each session consisting of 10 trials. During each trial, subjects were asked to focus on the flickering square for 10 s, followed by a 10 s rest interval. During each trial, subjects were instructed to refrain from blinking, moving, or talking. However, during rest, they were allowed to blink, move, and talk to ensure comfort. By considering the electrode connections, ensuring signal clarity, and conducting the experiment, the total duration of the experiment was approximately 30 min per subject. Figure 4 illustrates the step-by-step procedure followed in this research to achieve its objectives.

#### 3.3.1 EEG synchronization and trigger setup

**FIGURE 5:**
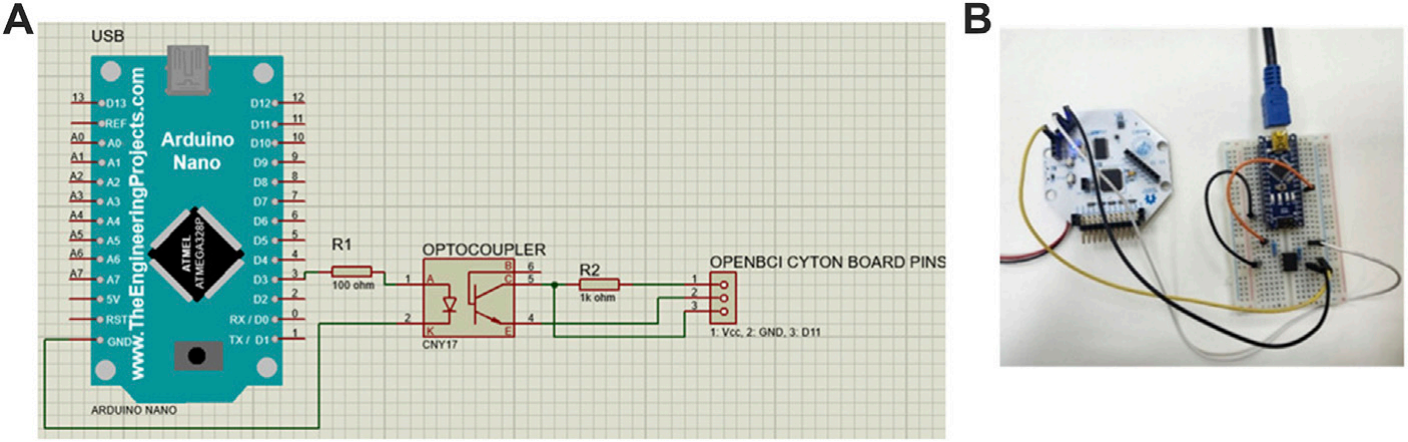
**(A)** Schematic connection of the external trigger in *Proteus* software. The Cyton board’s pins are simulated using a receptacle connector, with the three pins representing voltage (Vcc), ground (GND), and digital I/O pin (D11), respectively. The setup includes an Arduino Nano connected to an optocoupler to isolate and interface the external trigger with the Cyton board. **(B)** Real Circuit Connection. The Arduino Nano is powered via a USB connection to the computer.

The synchronization and precise timing between the stimulus and the data stream were essential to obtain accurate data. This was achieved by adding an external trigger to the OpenBCI Cyton board was the method used to achieve this. In this study, it was crucial to identify the EEG signal that occurs when the stimulation begins. The trigger was also used to distinguish between the rest and trial periods, which is essential for subsequent segmentation step. To accomplish this, the OpenBCI Cyton board mode was changed to digital mode in the OpenBCI GUI, allowing the reading to occur from the external pins. The electronic components used included a breadboard, an Arduino Nano, a CNY17 optocoupler, resistors (100Ω and 1 kΩ), and connecting wires. An optocoupler is a semiconductor device used to transmit electrical signals between two separate circuits. It consists of two parts: a light-emitting diode (LED) that emits light, and a light receiver, typically a photodiode or phototransistor. Both parts are hidden inside a body with metal legs for connection reasons (Storr, 2024). The visual representation of the electronic circuit used in the experiment is illustrated in Figure 5A, while the real circuit is depicted in Figure 5B.

The function of the circuit can be summarized as follow: First, stimulation starts once the “Start” button (shown in Figure 3) is clicked. Simultaneously, the Arduino output pin D3 of the Arduino sends a voltage. This voltage causes the LED in the optocoupler to emit light, activating the photodiode and forming a short circuit between pins 4 and 5 of the optocoupler to form a short circuit. This allows current to reach to the Cyton board as an external input. A wire connects pin 5 of the optocoupler to D11 of the Cyton board to enable this connection.

#### 3.3.2 Experimental design parameters

The experimental design parameters were carefully chosen to meet the objectives of the study. Firstly, some basic controlled laboratory conditions were considered in order to enhance the accuracy of the results. The experiments were conducted with consistent ambient lighting, maintaining a distance of approximately 30 cm from the stimulator, ensuring the invariability of environmental factors for all subjects. Design decisions also considered the participants” comfort, as the experiment should not exceed 30 min to avoid discomfort for the individuals undergoing the test. The flickering time was set to 10 s in each trial to avoid potential eye problems caused by the flickering effect. The rest period was also chosen to be 10 s to allow individuals to move and rest their eyes, ensuring their comfort. Additionally, more than 10 s could be a little challenging for individuals taking the test, as they may struggle to focus on the flickering for longer periods, potentially impacting the EEG data and leading to inaccuracies in the results. Important design parameters include the values of the frequencies used to elicit SSVEP, the colors of the background and flickering squares, the Ishihara plates used, and their specific arrangement. The selection of frequencies was particularly crucial, as they are fundamental in BCI systems based on SSVEP. Generally, these frequencies are categorized into three main ranges: the low band, the middle band, and the high band, which encompass the ranges of 5–12 Hz, 12–30 Hz, and 30–60 Hz, respectively (Zhang et al., 2017). In this study, the chosen frequencies were 15 Hz and 18 Hz, which fall within the middle band. These frequencies were selected based on previous research identifying the optimal values for eliciting SSVEP. In Duart et al. (2020), it was demonstrated that the middle range offers the best signal-to-noise ratio (SNR), indicating its advantages in SSVEP-based BCI systems. Additionally (Kuś et al., 2013), suggested that a specific range within the middle band (e.g., 12–18 Hz) is optimal for eliciting SSVEP. Furthermore (Zhang et al., 2017), found that the largest SSVEP amplitude occurs at a 15 Hz stimulus, making it a favorable choice for stimulation due to its easily recognizable amplitude during diagnosis. The second frequency, 18 Hz, was selected because it falls within the optimal range (Kuś et al., 2013) and offers a noticeable difference from 15 Hz, ensuring clear amplitude differentiation in SSVEP responses.

In addition to frequency selection, the design of the stimulus interface was also critical for obtaining accurate results. In this study, the background color was chosen as black, while the stimulation color for the flickering squares was selected as white. According to Duart et al. (2020), white and red were suggested as ideal colors when using middle-range frequencies. However, red color can be uncomfortable and may trigger epileptic seizures. Therefore, white was selected as the stimulation color. Another reason for choosing white is that the stimulation in this study lasts 10 s. As reported by Du and Zhao (2022), white color is found to be the best choice when the stimulation duration exceeds 1.5 s. Additionally, the black background was chosen because it outperforms other colors, such as red and blue, when paired with white stimulation (He and Abu Bakar, 2023). These color choices were made to maximize experimental efficiency, subject comfort, and result accuracy.

**FIGURE 6:**
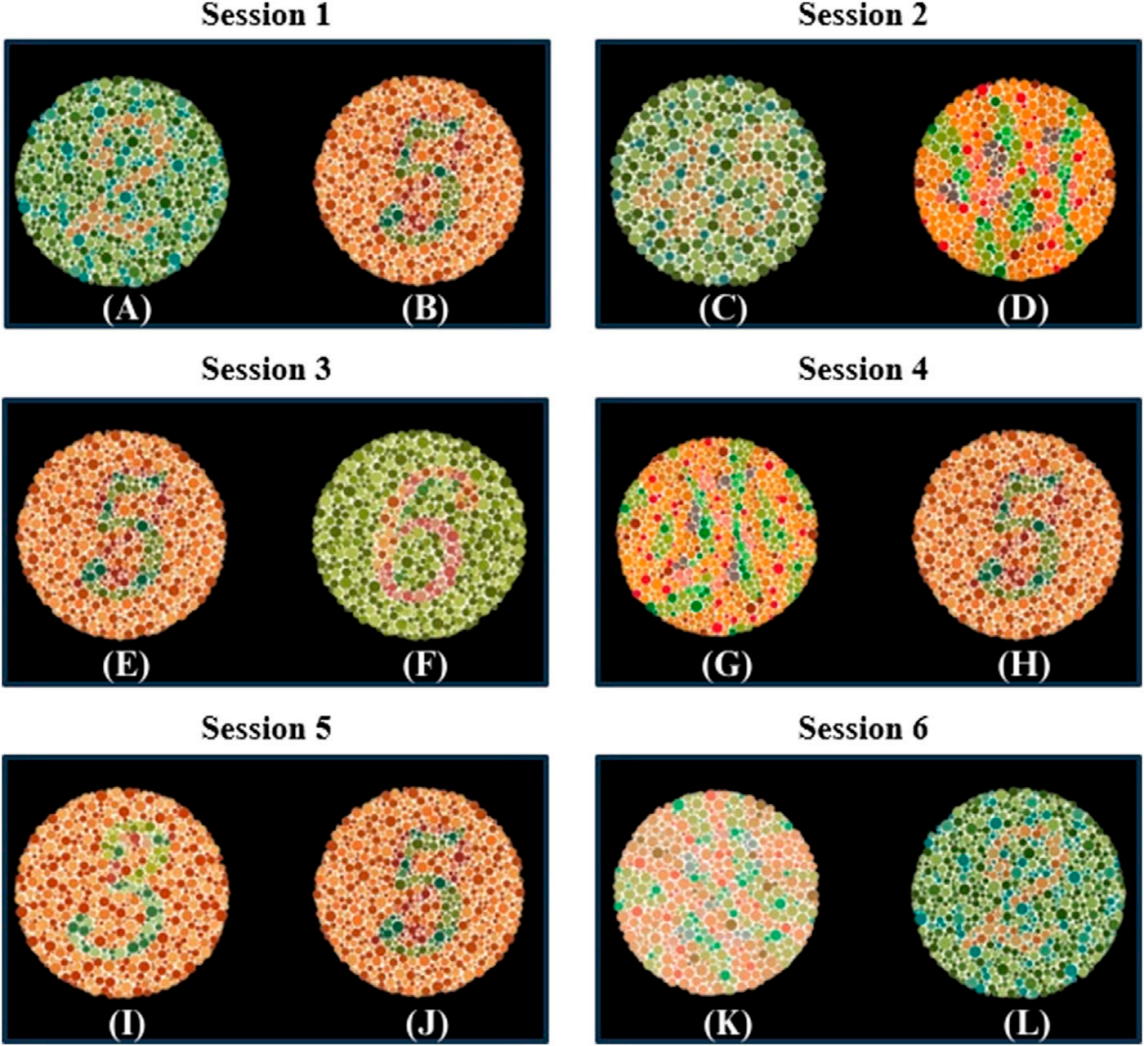
Ishihara plates pairs used in each Session. In each session, subjects were asked to choose between two plates. The target and non-target plates were as follows: Session 1 includes **(A)** the target plate showing “2” and **(B)** the non-target plate showing “5”; Session 2 includes **(C)** the target plate showing “45” and **(D)** the non-target plate showing a hidden digit plate; Session 3 includes **(E)** the target plate showing “5” and **(F)** the non-target plate showing “6”; Session 4 includes **(G)** the non-target plate showing a hidden digit plate and **(H)** the target plate showing “5”; Session 5 includes **(I)** the non-target plate showing “3” and **(J)** the target plate showing “5”; Session 6 includes **(K)** the non-target plate showing a hidden digit plate and (L) the target plate showing “2”.

A critical aspect of the experiment involved the subject’s selection between two plates based on a pre-identified number. For instance, if the subject is asked to choose the number “2”, they are expected to focus on the flickering squares below the plate they selected as number “2”. Thus, selecting appropriate plate pairs in each session is crucial for accurate diagnosis. To ensure the selection of appropriate plate pairs, 26 volunteers who suffer from red-green CVD participated in providing their answers for the standard Ishihara test (Colorlite, 2024). Their answers played a crucial role in determining the Ishihara plates used during the procedure. Another important factor considered was the presence of hidden digits on specific Ishihara plates. These hidden digits are designed so that individuals with normal vision are cannot recognize them, while those with red-green CVD can identify them (Birch, 1997). This is due to individuals with CVD relying on the S-cone, which detects short wavelengths and enables them to perceive blue and recognize the hidden digits (Miyahara, 2009). Importantly, the more hidden digits an individual can recognize, the more severe their CVD is (Birch, 1997). This phenomenon plays a significant role in assessing CVD severity. Figure 6 illustrates the Ishihara plates used, with the plates displaying hidden digits specifically shown in Figures 6D, G, K.

In this study, the design of each session with specific plate pairs was based on prior observations and theoretical expectations. In session 1, the “2” and “5” pair was selected because volunteers consistently perceived “5” as “2”, while the “2” appeared as nothing. This observation aligns with the findings presented in (Ekhlasi et al., 2021), which demonstrated that individuals with red-green CVD perceive “2” shown in Figure 6A as nothing. In session 2, the pair consists of “45” and one of the hidden digit plates shown in Figure 6D, which represents “nothing”. It is expected that individuals with CVD recognize the “nothing” plate shown in Figure 6D as “45”(Hidden plates, 2024). In session 3, the pair consists of “5”, which is recognized by volunteers with CVD as “2”, and “6”, which is recognized by the volunteers as “5”. This arrangement allows for diagnosis since individuals with CVD will choose “6” if asked to identify “5”, unlike individuals with normal vision who will identify it correctly.

In session 4, the pair consists of the hidden digit plate shown in Figure 6G, which represents “nothing”, and the other plate is “5”. The hidden digit plate shown in Figure 6G was recognized by volunteers as “5”. This aligns with (Ekhlasi et al., 2021), where CVD subjects identified this specific hidden digit as “5”. This ensures that when a CVD individual is asked to identify “5”, they will choose the hidden digit shown in Figure 6G instead. In session 5, the pair consists of “3”, which is recognized “5” by some volunteers, while others perceive it as “nothing” or “3”. The other plate is “5”, which is recognized as “2” by individuals with CVD. This session may serve as an indicator of CVD severity, as some individuals may correctly identify it. Finally, session 6 involves a hidden digit shown in Figure 6K, expected to be recognized as “2”(Hidden plates, 2024), and a “2”, typically perceived as “nothing” by individuals with CVD (Ekhlasi et al., 2021).

Theoretical expectations were also developed to predict the selections of individuals with CVD based on the chosen plates. The selection of these plates pairs in the sessions is designed to accurately diagnose CVD. Individuals with CVD are expected to choose specific plates that differ from those selected by individuals with normal vision. Each of the six sessions contains a different pair of plates, ensuring the accuracy of the diagnosis. Even if a subject guesses an answer in one session, the likelihood of consistent guessing is reduced as the plates vary from session to another. The theoretical expectation of subjects’ selections based on the plates are illustrated in Table 2.

**TABLE 2:** Selection expectations based on questions in each session.

Sessions #	Question in each session	Normal vision selection	CVD selection
1	Which one is number 2?	Figure 6A	Figure 6B
2	Which one is number 45?	Figure 6C	Figure 6D
3	Which one is number 5?	Figure 6E	Figure 6F
4	Which one is number 5?	Figure 6H	Figure 6G
5	Which one is number 5?	Figure 6J	Figure 6I
6	Which one is number 2?	Figure 6L	Figure 6K

### 3.4 EEG data pre-processing and feature extraction

The raw EEG data were collected using the OpenBCI GUI software (version 5.2.2), saved as. txt files, and then imported into Python and MATLAB for processing. First, the EEG data for all subjects were filtered using a 4th-order Butterworth bandpass filter (BPF) with a cutoff frequency range of 5–30 Hz, followed by a notch filter to eliminate 60 Hz powerline noise, both applied in the OpenBCI GUI software. Additionally, a second BPF with a range of 5–50 Hz was applied in Python to ensure better signal representation. Next, the EEG data were re-referenced to the average of all channels to improve the SNR. The data were then segmented into 10-s trials for each session. After segmentation, artifact rejection was performed using Independent Component Analysis (ICA) to remove artifacts-related components, such as eye-blinks, from the EEG signals. After the ICA-based removal process, visual inspection and amplitude-based rejection were conducted to discard trials in which any EEG channel exceeded ±100 μV. After that the 10 trials for each session were averaged to improve the SNR.

The SSVEP data segmentation was performed using Python. First, during data recording, a trigger was utilized to separate the data into two distinct conditions as previously mentioned. When the subject focused on the stimulation, the trigger was set to 0; conversely, when the subject was not stimulated, the trigger was set to 1 in each session. The data was segmented into 10-s intervals, alternating between “trial” (stimulation) and “rest” (no stimulation) segments. This structured segmentation approach facilitated data organization for subsequent analysis. With 10 trial and 10 rest periods in each session, the averages of these segments were calculated separately for each session and used in the classification process.

Feature extraction was conducted using traditional target identification algorithms, including fast Fourier transform (FFT), power spectral density (PSD), and canonical correlation analysis (CCA). PSD, a technique widely employed for detecting SSVEPs, allows for the extraction of frequency information from EEG signals. PSD values were computed using the Welch method with a Hann window to mitigate sharp transition effects on frequency content, and the results were subsequently converting to µV^2^/Hz. The 10-s EEG data windows (2,500 samples) were divided into 19 segments (250 samples) with a 50% overlap. On the other hand, CCA, known for its rapidity and straightforward integration, was utilized to identify target frequencies. CCA facilitates the identification of the target stimulation frequency through correlation coefficient analysis by constructing reference signals using sine and cosine templates. These methods were utilized for their efficacy in handling multichannel EEG signals and optimizing electrode and time parameters for SSVEP analysis (Hakvoort, 2010; Scarpino et al., 1990; Ma et al., 2022).

For PSD, the equation for the periodogram of each segment is given by Welch’s Method (2024), as shown in Equation 1.

$$P\left(k\right)=\frac{{\left|X\left(k\right)\right|}^{2}\;}{M}$$ (1)

where $P\left(k\right)$ represents the periodogram at frequency k , ${\left|X\left(k\right)\right|}^{2}$ denotes the magnitude squared of the Fourier transform of the signal at frequency k , and M represents the total number of samples in the signal.

The Welch estimate of the power spectral density is then given by Welch’s Method (2024), as shown in Equation 2.

$$S\left(k\right)=\frac{1}{N}\times \sum \left[P\left(n\right)\left(k\right)\right]$$ (2)

where $S\left(k\right)$ represents the Welch estimate of the PSD at frequency k , N represents the number of frames used in the Welch method. $\left.P\left(n\right)\left(k\right)\right]$ represents the periodogram of the nth frame at frequency k . This equation calculates the average of all the periodograms at frequency k .

CCA involves examining two sets of variables $\left(X,Y\right)$ . Unlike other correlation methods, CCA is capable of detecting robust linear relationships between multidimensional variables that might go unnoticed due to the coordinate system used. CCA’s effectiveness lies in identifying pairs of linear transformations ( $\left.W_{x},W_{y}\right)$ for these variable sets. These transformations maximize the correlation between the coordinate systems when applied (Hakvoort, 2010; Wang et al., 2014).

The resulting projections of these transformations are termed canonical variates and can be expressed as shown in Equation 3 (Hakvoort, 2010).

$$\left(W_{x},W_{y}\right)=\mathrm{argmax}\left|corr\left(XW_{x},YW_{y}\right)\right|$$ (3)

where $W_{x}$ and $W_{y}$ represent the linear transformations, and X and Y represent the sets of variables.

CCA was performed for each stimulation frequency between a set of EEG signals in X and a set of $Y_{f}$ of SSVEP responses, as shown in Equation 4 (Hakvoort, 2010).

$$Y_{f}=\left(\begin{array}{c} \sin\left(2\pi ft\right)\\ \cos\left(2\pi ft\right)\\ .\\ .\\ .\\ \sin\left(2\pi Hft\right)\\ \cos\left(2\pi Hft\right) \end{array}\right)$$ (4)

Where f represents the stimulation frequency, H represents the number of harmonics, and T represents the sampling period, and $f_{s}$ represents the sampling rate. While CCA produces several correlation coefficients, the largest one was considered. In this study, the first 3 harmonics were included in the CCA analysis to capture the strongest components of the SSVEP response, as higher harmonics generally have lower SNRs and introduce more noise.

To analyze the neural responses, a two-way repeated measures ANOVA was performed using SPSS Statistics (IBM, Armonk, NY, United States), with frequency (15 Hz and 18 Hz) as a within-subjects factor and participant groups (normal vs CVD) as a between-subjects factor (Park et al., 2009). The statistical significance level (α) for all analyses was set at *p* ≤ 0.05. Mauchly’s test of sphericity was applied to verify the assumption of sphericity for the within-subjects comparisons, and Greenhouse-Geisser or Huynh-Feldt corrections were applied when necessary to adjust for any violations.

### 3.5 Data augmentation for balanced features using SMOTE

For each subject, a feature matrix was generated from the EEG data, comprising PSD and CCA features. From each selected EEG channel (O1, O2, Cz, and Pz) and target frequencies (15 Hz and 18 Hz), a total of 16 features per subject were extracted. Specifically, this feature set included 8 features from PSD (4 channel × 2 frequencies) and 8 features from CCA (4 channel × 2 frequencies) to efficiently capture relevant characteristics of EEG signals for distinguishing between healthy and CVD subjects. Given the class distribution, the feature matrix contained a total of 176 features for healthy subjects (16 features × 11 subjects) and 80 features for CVD subjects (16 features × 5 subjects).

To address this imbalance and improve classification performance, Synthetic Minority Over-sampling Technique (SMOTE) was implemented to balance the class distribution in our data (Chawla et al., 2002). SMOTE is a widely used oversampling algorithm that proportionally increases the representation of minority classes, thereby balancing the number of samples in each class. In this study, ensuring that the classifier model is trained on an equal number of samples from each class is essential to avoid model bias toward the majority class. To achieve this balance, SMOTE was applied to augment the minority class data (CVD) with synthetic samples. SMOTE was implemented on the combined feature set, generating synthetic samples for the minority class by interpolating between existing PSD and CCA values, resulting in an equal number of samples for each class in the training set. The SMOTE algorithm generates these synthetic samples using the following (Equation 5).

$$x_{new}=x_{i}+\lambda \left(x_{j}-x_{i}\right)$$ (5)

where $x_{new}$ is the new synthetic sample generated, $x_{i}$ is an existing sample from the minority class, $x_{j}$ is a randomly chosen nearest neighbor of $x_{i}$ within the minority class, and λ is a randomly selected value between 0 and 1 for each synthetic sample, which determines the position of $x_{new}$ along the line segment connecting $x_{i}$ and $x_{j}$ .

### 3.6 Classification procedures

**FIGURE 7:**
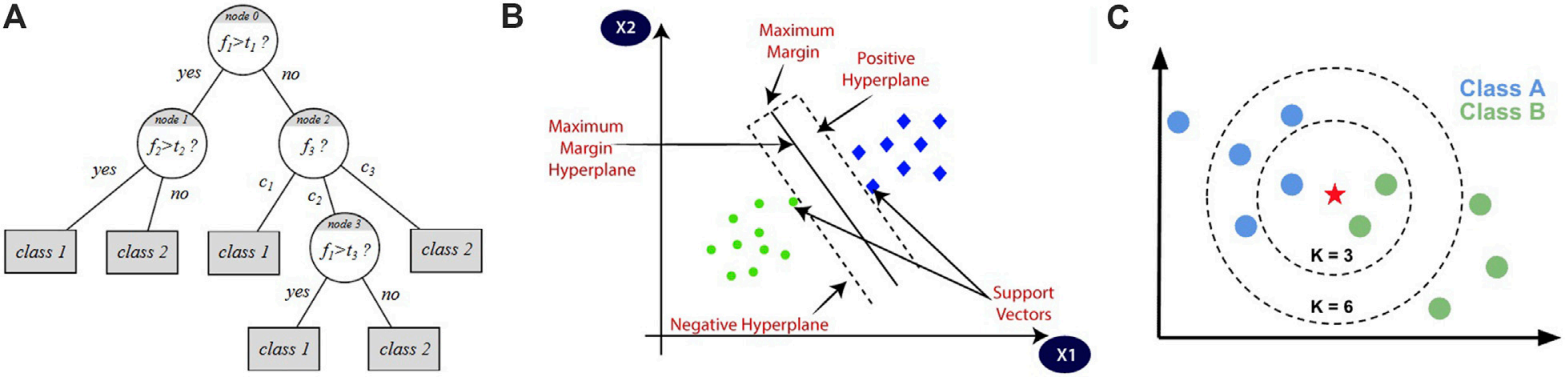
Illustrations of the three classification algorithms: **(A)** represents DT algorithm, **(B)** represents SVM algorithm, and **(C)** represents KNN algorithm (Chawla et al., 2002).

After balancing the dataset using SMOTE, we applied three distinct classifiers (Decision Tree (DT), Support Vector Machine (SVM), and K-Nearest Neighbors (KNN)) to assess their performance in differentiating CVD from healthy cases. The DT classifier creates models to classify data by representing decision logics. This algorithm takes the form of a tree-like structure, comprising multiple levels, with the top node referred to as the root node. The internal nodes of the tree correspond to tests conducted on input variables, and based on the outcomes of these tests, the algorithm branches out to the appropriate nodes. The leaf nodes of the tree represent the decision outcomes. Building decision trees depends on calculating the entropy based on the data requirements (Uddin et al., 2019; Decision Tree, 2024). Figure 7A shows an illustration of the DT algorithm.

The equation that represents the entropy of a single attribute is shown in Equation 6 (Britannica, 2024).

$$E\left(S\right)=\sum_{i=1}^{c}p_{i}\log_{2}p_{i}$$ (6)

Where $E\left(S\right)$ represents the entropy function, $p_{i\;}$ represents the probability of an event S .

The SVM classifier has the ability to classify data depending on the Kernel method, which utilizes a linear classifier for non-linear problems, making it capable of classifying both linear and non-linear data. It transforms each data point into a different space and identifies a hyperplane that effectively separates the data into different classes. By representing each data point as a coordinate in space, the SVM algorithm determines the optimal hyperplane to distinguish the classes (Uddin et al., 2019; Boateng et al., 2020). Figure 7B shows an illustration of the SVM algorithm.

The equation that represents the SVM classifier is given by Equation 7.

$$K\left(x_{i,}x_{i}^{\prime }\right)={\left(1+\sum_{j=1}^{p}x_{ij}x_{ij}^{\prime }\right)}^{\wedge }d$$ (7)

Where K represents the Kernel function, $\left(x_{i,}x_{i}^{\prime }\right)$ represents the inner products of the training observations, and p represents the degree of the polynomial kernel.

The KNN classifier depends on the number of nearest neighbors, denoted as K . By choosing different values for K , the classification results for a given sample object will vary. The Euclidean distance function is used in the measurement process to assign the case to the nearest neighbor class (Uddin et al., 2019; Boateng et al., 2020). Figure 7C shows an illustration of the KNN algorithm, where the star represents the new object that is classified as black when K=3 , while it is classified as red when K=5 .

The equation that represents the Euclidean distance metrics used in the KNN classifier is given by Equation 8 (Boateng et al., 2020).

$$D\left(x,y\right)=\sum_{i=1}^{N}\sqrt{x_{i}^{2}\_{\;y}_{i}^{2}}$$ (8)

where $D\left(x,y\right)$ represents the distance between the points x and y , and N represents features numbers.

Moreover, the classification conducted in this study was evaluated using various methods. Initially, classification accuracy is a common method to evaluate the performance of classifying EEG signals in BCI applications. We used *k*-fold cross-validation to train and test extracted features for all classifiers, where *k* was set to 5. In this approach, the dataset was divided into 5 partitions, with 4 partitions iteratively used for training and the remaining partition used to test the model’s performance. The accuracy can be calculated using Equation 9.

$$Accuracy=\frac{Number\;of\;correct\;SSVEP\;responses\;assigned\;classes\;}{Total\;number\;of\;SSVEP\;responses}\times 100$$ (9)

To analyze the classification accuracies, - a two-way repeated measures ANOVA was performed using SPSS Statistics (IBM, Armonk, NY, United States), with classifier type (SVM, DT, KNN) and feature set (PSD, CCA, PSD + CCA) as within-subjects factors. The statistical significance level α for all analyses was set by p≤0.05 .

The confusion matrix provides a comprehensive evaluation of the classification model’s performance by offering essential data to calculate precision, recall, and the F1-score, which collectively provide a complete picture of the model’s performance. The matrix consists of different situations (illustrated in Table 3), which are True Positive (TP), True Negative (TN), False Positive (FP), and False Negative (FN) for the three classes.

**TABLE 3:** Confusion matrix situations.

Situation	Meaning
True Positive (TP)	Represents the number of instances that belong to the CVD class and are correctly classified as CVD.
True Negative (TN)	Represents the number of instances that belong to the normal (healthy) class and are correctly classified as normal
False Positive (FP)	Represents the number of instances that belong to the normal class but are incorrectly classified as CVD.
False Negative (FN)	Represents the number of instances that belong to the CVD class but are incorrectly classified as normal

The F1 score can be calculated by first computing the precision and recall using Equations 10–12.

$$Precesion=\frac{TP\;}{TP+FP}$$ (10)

$$Recall=\frac{TP\;}{TP+FN}$$ (11)

$$F1-score=\frac{2\times Precesion\times Recall\;}{Precesion+Recall}$$ (12)

It is worth noting that precision is also referred to as the positive predictive value, recall is referred to as sensitivity or true positive rate, while specificity is referred to as the true negative rate. Also, sensitivity is associated with TP and FN, while specificity is associated with TN and FP.

## 4 Results

This section presents the outcomes and insights derived from the research. It includes the findings, interpretations, implications, and potential limitations. Additionally, it evaluates the methodology by presenting the results and comparing them with other studies.

### 4.1 SSVEP response

**FIGURE 8:**
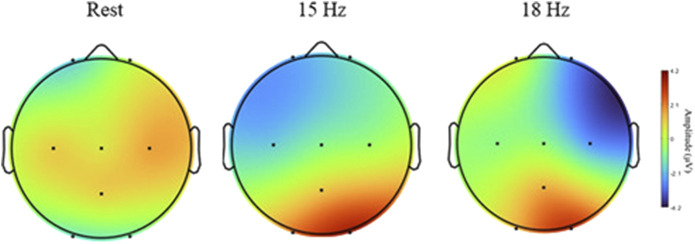
Topographical maps of SSVEP amplitudes of different conditions: rest, 15 Hz, and 18 Hz. Brain activity is shown across eight electrodes (O1, O2, PZ, C3, CZ, C4, FP1, FP2).

SSVEP responses were observed throughout the experiment. Figure 8 illustrates the topographical maps of SSVEP amplitudes under three different conditions: rest, 15 Hz stimulation, and 18 Hz stimulation. These maps display the distribution of the brain activity across the scalp recorded from eight electrodes (O1, O2, Pz, C3, Cz, C4, Fp1, Fp2) placed according to 10–20 system. During the rest condition, the SSVEP activity is predominantly low across most of the scalp, particularly in parietal and occipital regions. However, the central region exhibits a higher amplitude, as indicated by the red coloration in the topographical map. This observation suggests that the brain maintains a certain level of baseline activity even without external visual stimuli. This persistent activity could be attributed to several factors, including alpha wave generation, spontaneous brain processes, and the influence of Default Mode Network (DMN) (Knyazev et al., 2011).

At 15 Hz stimulation, the topographical map reveals a notable increase in SSVEP amplitude, particularly in the occipital region (O1 and O2). This finding aligns with expectations, given that these areas of the brain are responsible for visual processing. The heightened amplitude in these areas indicates that the 15 Hz visual stimulus generates a strong neural response in the visual cortex. Furthermore, the map shows that while the activity is concentrated in the occipital region, it also extends into adjacent parietal areas, suggesting a broad activation of the brain’s visual processing networks when exposed to the 15 Hz frequency. As SSVEPs are primarily generated in the occipital lobe, a relative reduction in activity in the frontal and central regions is observed, as depicted in Figure 8. Similarly, during 18 Hz stimulation, a clear increase in SSVEP amplitude, particularly in the occipital region (O1 and O2), was observed. These observations suggest that the 15 Hz and 18 Hz frequencies effectively elicit strong neural responses and robustly stimulate the visual cortex, making them ideal choices for SSVEP-based CVD diagnostic.

**FIGURE 9:**
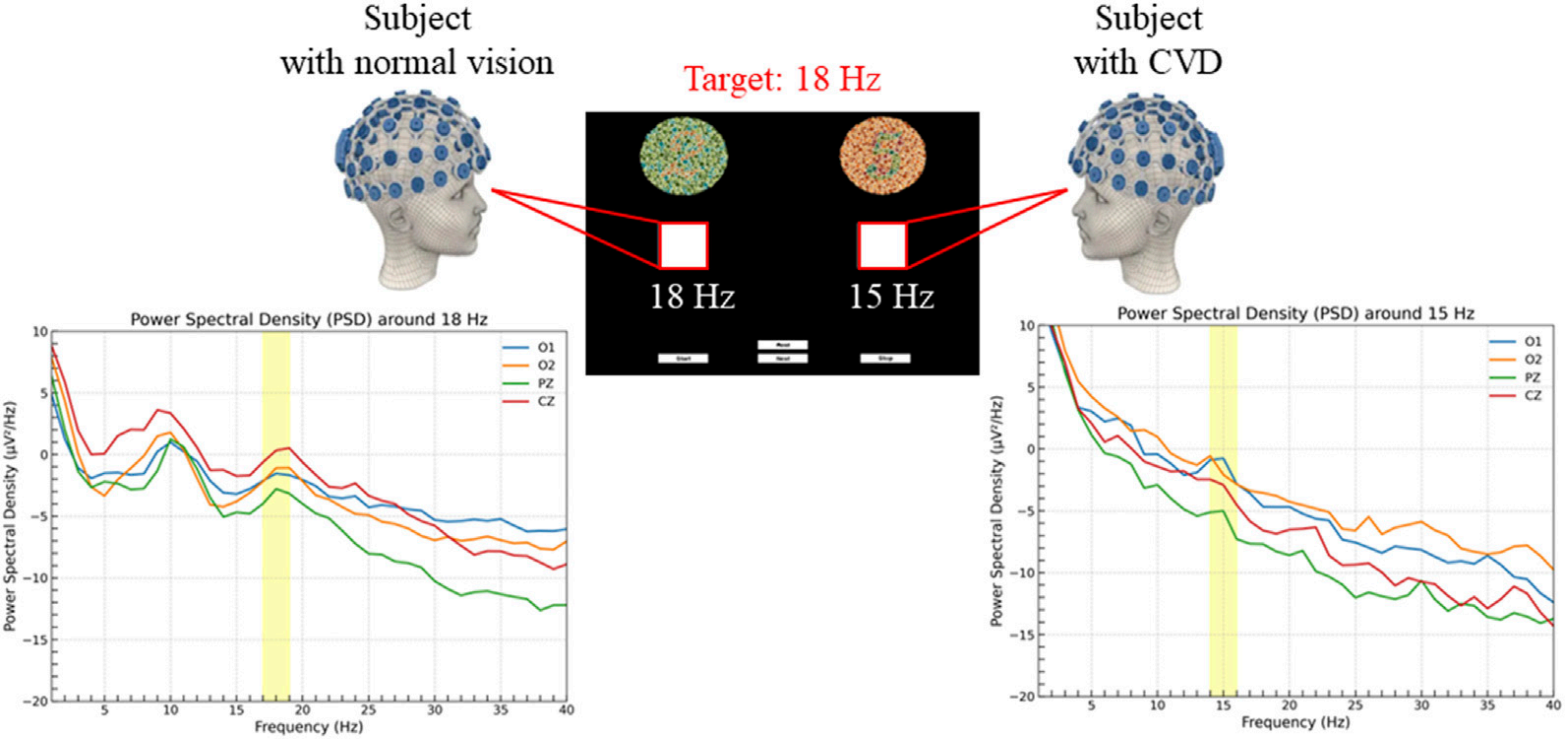
PSD analysis in subjects with normal vision (left) and color vision deficiency (CVD) (right) during the experiment. Both subjects were instructed to focus on a target square flickering at 18 Hz, corresponding to the number “2”. The subject with normal vision correctly identified the 18 Hz target, resulting in a distinct PSD peak around 18 Hz, while the subject with CVD incorrectly identified the target, resulting in a PSD increase around 15 Hz associated with the number “5”.

**FIGURE 10:**
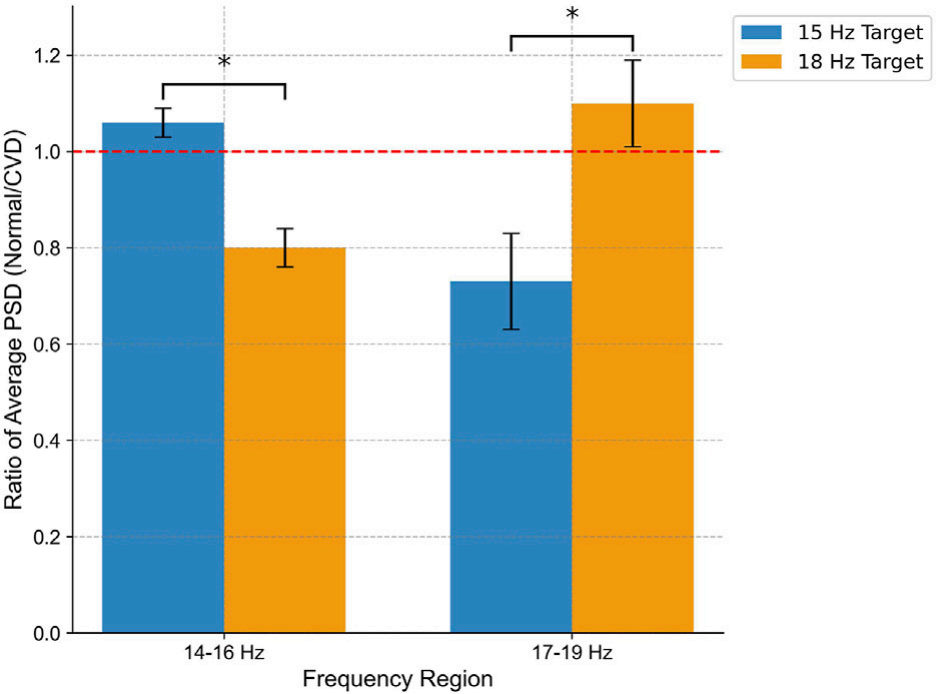
Comparison of average PSD ratio between normal vision and CVD subjects in two frequency regions (14–16 Hz and 17–19 Hz) for flickering targets at 15 Hz (blue) and 18 Hz (orange) measured at channel O2. The PSD ratio is determined by dividing the average PSD in individuals with normal vision by that in individuals with CVD. Notably, normal vision subjects show enhanced neural entrainment to 15 Hz in the 14–16 Hz range and to 18 Hz in the 17–19 Hz range, while CVD subjects exhibit the opposite pattern. Statistical Significance (*p* < 0.05) underscores distinct neural processing dynamics.

The SSVEP responses were analyzed using four electrodes (O1, O2, Pz, Cz) which exhibited the highest activity during stimulation and are particularly significant for SSVEP analysis. Figure 9 shows the PSD analysis of SSVEP responses for two subjects: one with normal vision and another with CVD. The central portion of the figure presents the visual stimuli, with the target frequency set at 18 Hz. The graphs on either side illustrate the PSD results for the occipital electrodes (O1, O2) and central electrodes (Pz, Cz), providing a comprehensive view of the neural responses recorded from these specific electrode locations. This detailed analysis allows for a deeper understanding of how individuals with normal vision and CVD exhibit distinct brain activity patterns in response to visual stimuli, particularly in relation to the target frequencies presented. For the subject with normal vision, the PSD graph on the left reveals a distinct peak around 18 Hz, particularly evident in the occipital regions (O1 and O2). This robust neural response signifies that the subject’s brain is effectively synchronized with the 18 Hz stimulus, aligning precisely with the intended target frequency for focus and attention. In contrast, for the subject with CVD, the PSD graph on the right displays a peak response around 15 Hz instead of the target 18 Hz. This indicates that the subject focused on the 15 Hz, despite the 18 Hz stimulus being the target. This misalignment underscores the difficulties individuals with CVD encounter in accurately processing visual stimuli, shedding light on the distinct challenges they face in cognitive processing and perception. A distinct peak at 10 Hz (non-target) was observed for both stimulation frequencies but becomes more apparent when the subject focuses on 18 Hz. Moreover, upon comparing the average PSD ratios between normal vision and CVD subjects, the differences in their responses are evident in Figure 10. Ratios greater than 1 signify higher PSD in normal vision subjects, whereas ratios below 1 indicate higher PSD in CVD subjects. The PSD ratio is calculated as the average PSD in normal vision subjects divided by that in CVD subjects. In the 14–16 Hz range, normal vision subjects exhibit higher PSD for the 15 Hz target, whereas CVD subjects display higher PSD for the 18 Hz target. Conversely, in the 17–19 Hz range, normal vision subjects demonstrate higher PSD for the 18 Hz target, while CVD subjects showcase higher PSD for the 15 Hz target.

To examine the observed differences in neural responses, a two-way repeated-measures ANOVA was performed using color vision status (normal vs CVD) and frequency region (14–16 Hz vs 17–19 Hz) as factors. The results revealed a significant interaction effect between color vision status and frequency region on the average PSD ratio $\left.\left(F\left(1.14\right)=5.76,p=0.021\right)\right)$ as shown in Figure 10. This finding indicates that the effect of color vision status on the PSD ratio differs depending on the frequency region being analyzed. Bonferroni-corrected *post hoc* comparisons further showed that normal vision subjects exhibited significantly higher PSD ratios at the 15 Hz target in the 14–16 Hz range and at the 18 Hz target in the 17–19 Hz range compared to CVD subjects, reflecting a potential difference in visual perception at these frequencies.

### 4.2 Classification results

**FIGURE 11:**
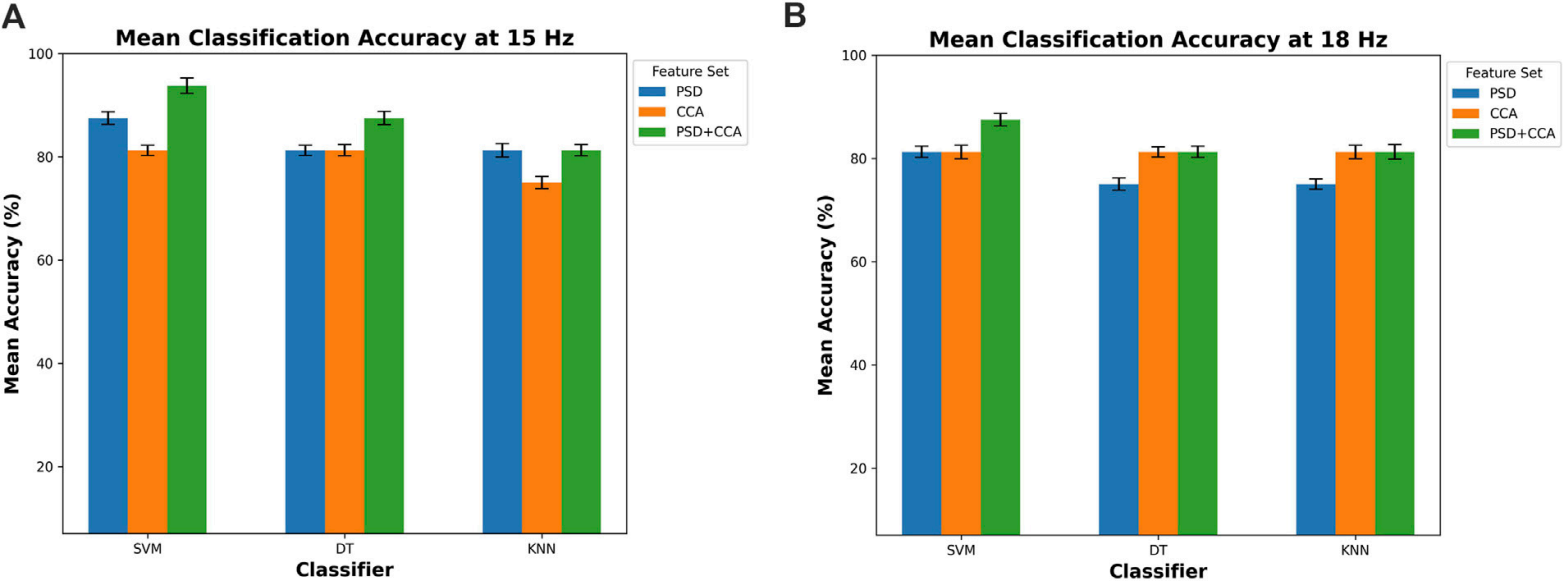
Mean classification accuracy for **(A)** 15 Hz and **(B)** 18 Hz across three classifiers (DT, SVM, and KNN) using different feature sets (PSD, CCA, and PSD + CCA).

The classification accuracy is shown in Figure 11, offering a detailed insight into the performance of three distinct classifiers: DT, SVM, and KNN. These classifiers are evaluated across two pivotal target frequencies, which are 15 Hz and 18 Hz, providing a nuanced understanding of their efficacy at different frequency levels. Moreover, the evaluation is conducted using three feature sets: PSD, CCA, and a combined feature set comprising both PSD and CCA (PSD + CCA). This approach allows for a thorough examination of how these classifiers perform when leveraging distinct feature sets, shedding light on the interplay between feature selection and classification accuracy. This detailed analysis provides valuable insights into the optimal configurations for achieving high accuracy in this classification task. Each classifier is represented by three bars corresponding to the different feature sets, with error bars indicating the standard deviation.

**FIGURE 12:**
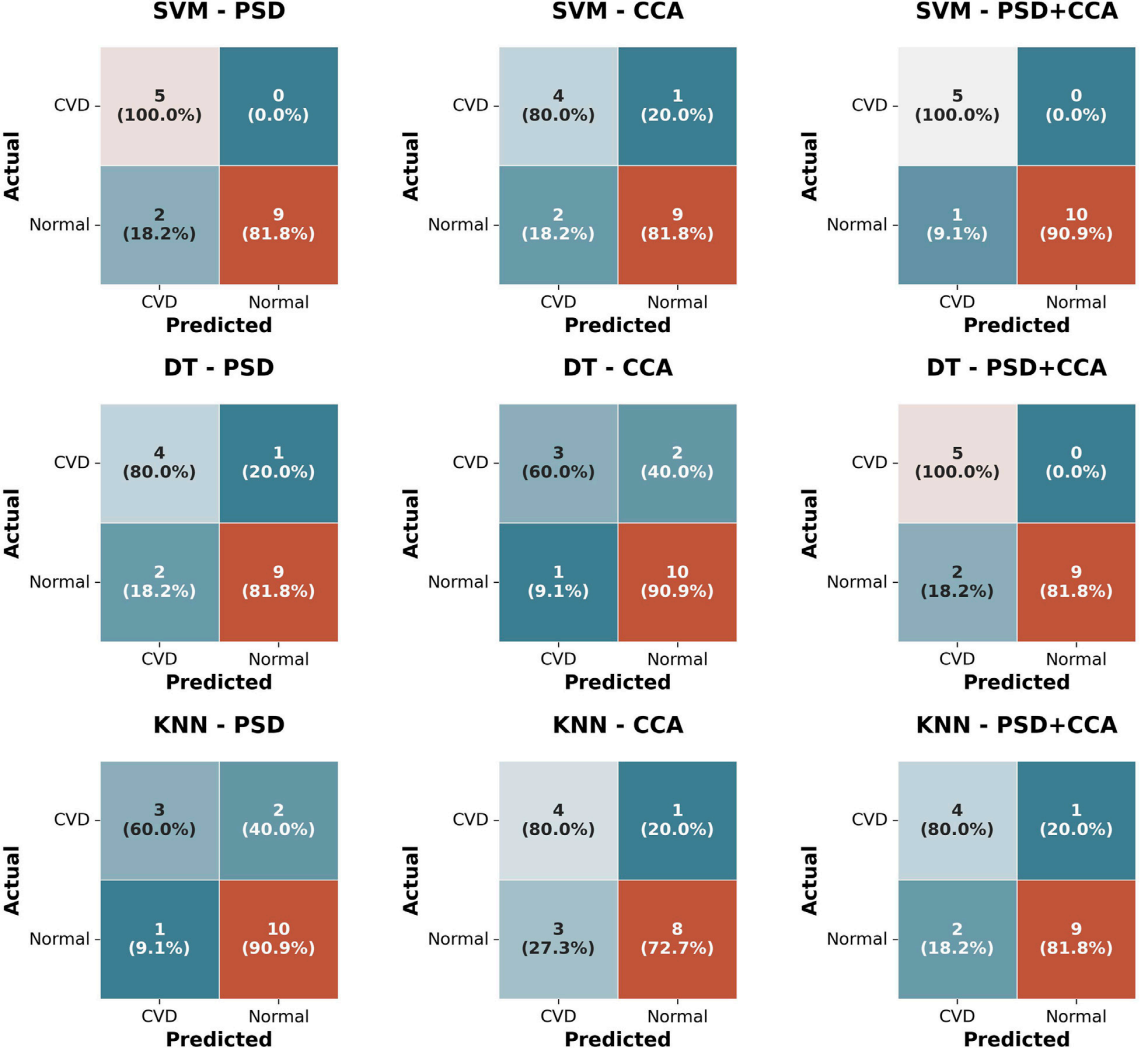
Confusion matrices for three Classifiers (SVM, DT, KNN) using three feature sets (PSD, CCA, PSD + CCA) for classifying normal vision and CVD subjects.

In both conditions at 15 Hz and 18 Hz, the classifiers consistently achieve high accuracy levels, exceeding the 75% threshold. This indicates the effectiveness of the classifiers in accurately classifying data based on the chosen feature sets. At 15 Hz, depicted in Figure 11A, the SVM classifier demonstrates the highest accuracy, reaching 93.75% ± 1.5% with the combined feature set (PSD + CCA), notably outperforming the individual feature sets (PSD and CCA). A similar trend is observed with the DT classifier, which achieves an accuracy of 87.5% ± 1.3% with PSD + CCA, slightly below that of SVM. For the KNN classifier at 15 Hz, both the PSD and PSD + CCA feature sets yield the same accuracy of 81.25%, indicating limited performance for KNN with feature set combination.-When analyzing the results at 18 Hz (Figure 11B), it is evident that the SVM classifier outperforms both DT and KNN across most of feature sets, achieving an accuracy of 87.5% ± 1.3% with PSD + CCA feature set. This consistent superior performance of SVM at 18 Hz suggests its effectiveness in accurately classifying data at this specific frequency. Additionally, DT and KNN classifiers exhibit similar performance, at around 81.25% with the same feature set, though SVM maintains a clear accuracy advantage - when using PSD and PSD + CCA feature set. Overall, the highest accuracy is achieved by the SVM classifier using the combined feature set (PSD + CCA) for both frequencies.-The exceptional performance of SVM when combining multiple feature sets can be attributed to its proficiency in handling high-dimensional feature spaces effectively, a characteristic for which SVM is renowned (Cervantes et al., 2020). A two-way repeated-measures ANOVA was conducted with classifier type (SVM, DT, KNN) and feature set (PSD, CCA, PSD + CCA) as within-subjects factors. The analysis revealed no significant main effect of classifier type on classification accuracy $\left(F\left(2,28\right)=1.34,p=0.28\right),$ indicating that accuracy levels did not differ significantly among the classifiers. Similarly, no significant main effect of feature set was observed $\left(F\left(2,28\right)=0.92,p=0.41\right.$ ), suggesting that the choice of feature set (PSD, CCA, or PSD + CCA) did not significantly impact classification accuracy. Moreover, based on the confusion matrices presented in Figure 12, the SVM classifier shows more accurate predictions and fewer misclassifications, particularly when using the combined feature set (PSD + CCA). This is shown by the high rates of correct classification, with SVM achieving 100% accuracy for CVD cases and 90.9% for normal cases using PSD + CCA, demonstrating its effectiveness in distinguishing between the two classes.

## 5 Discussion

In this study, a novel non-invasive method for diagnosing CVD was introduced, utilizing SSVEP feature extraction and classification. Subjects were exposed to varying frequencies to trigger the SSVEP response, leading to successful data analysis and classification. This method offers universal applicability, accommodating individuals facing mobility or communication challenges. Unlike traditional approaches, this method requires no extensive training, ensuring time efficiency and objective results. By sidestepping behavioral responses, training requirements, subjectivity, and potential manipulation of results, this method outperforms existing diagnostic techniques.

The spatial characteristics depicted in Figure 8, show high activity in the occipital and parietal electrodes (O1, O2, Pz) during the stimulation period (15 Hz, 18 Hz), which are typically the most affected areas during SSVEP elicitation. The activation of occipital and parietal regions during color vision can be elucidated by considering the neural pathways involved in processing color information. The occipital lobe, known for its role in visual processing since it contains the primary visual cortex that receives input from the lateral geniculate nucleus (LGN) located in the thalamus. The processing of color information involves the activation of cone cells in the retina, which are sensitive to different wavelengths of light corresponding to different colors. These signals travel through the optic nerve to the LGN and then to the visual cortex, where complex processing occurs (ScienceDirect Topics, 2024; Covington and Al Khalili, 2024). On the other hand, the parietal lobe is involved in integrating sensory information from various modalities, including vision. It plays a role in spatial perception, and the coordination of visual-motor tasks. During color vision tasks, the parietal cortex may be engaged in processes related to spatial awareness of stimuli, object recognition based on color cues, and the coordination of motor responses to visual color information (Souza-Couto et al., 2023). So, the heightened activity in these brain areas during SSVEP tests at 15 Hz and 18 Hz likely indicates that the brain is actively processing color information. This activity reflects the complex neural pathways responsible for understanding and reacting to colors in our environment.

Additionally, the activity in the parietal electrode (Pz) surpasses that of the central electrode (Cz). These findings are in line with previous research, such as (Norcia et al., 2015), which suggests that the SSVEP generates its most robust responses in occipital areas, originating from the visual cortex in the occipital region responsible for visual processing. Moreover, as highlighted in (Srinivasan et al., 2006), optimal SSVEP responses are attainable in both occipital and parietal regions. These studies align with the results of this study, as heightened activity was observed in the parietal and occipital regions, resulting in increased amplitudes and stronger SSVEP responses. The clarity of the proposed diagnostic technique relies on the activity differences observed in these regions. This finding helps explain the channel selection in this study, suggesting that these electrodes are the most suitable choices when measuring SSVEP responses. Furthermore, another study (Nguyen et al., 2019) revealed that the SSVEP responses were weakest and had the smallest amplitudes in central regions compared to occipital. This finding may explain the lower activity observed in the central electrode in this study, suggesting that the central channels may not be the optimal choice for diagnosing CVD using SSVEP measurements. However, it is important to note that the strength of SSVEP responses is influenced by the flickering frequencies used.

Furthermore, as expected, during the experiment, all five CVD subjects failed to choose the correct options in all sessions, confirming the selection expectations of the subjects” answers presented in Table 2 with a 100% success rate. Each of the five CVD subjects consistently selected the Ishihara plate corresponding to the non-target frequency, indicating that they incorrectly identified the numbers and confirming their CVD condition. This ensures the proper arrangement of Ishihara plate pairs and their ability to successfully distinguish between individuals with normal vision and those with CVD, as the arrangement process in this research involves selecting pairs that may be intertwined for those suffering from CVD. In Figure 9, the PSD exhibits a peak around 18 Hz for normal vision subject, ensuring that the subject was focusing on the target frequency, which means they chose the Ishihara plate correctly. Conversely, the subject with CVD incorrectly identified and focused on a square flickering at 15 Hz, associated with the number “5”, resulting in a PSD increase around 15 Hz. However, for both stimulating frequencies, there was a clear peak that appeared at approximately 10 Hz for most subjects. This finding is actually consistent with what was reported in Xie et al. (2016) and Kuś et al. (2013). In Xie et al. (2016), they observed a peak at 10 Hz, which was a non-target frequency, while the target frequencies were 8 and 12 Hz. Similarly, in this study, a peak appeared at 10 Hz when the target frequencies were 15 and 18 Hz. The reason for this could be explained by what was discussed in Xie et al. (2016) as they suggested that this peak may appear due to the gray-to-gray (GTG) effect, which depends on pixels (Ryans Computers, 2024) and the screen characteristics. These factors are difficult to eliminate. Additionally, in Kuś et al. (2013), they also observed a sharp peak at 10 Hz when the stimulation frequency was in the range of 13–25 Hz. They suggested that this occurrence could be attributed to findings from previous research by Regan (1975), where they demonstrated that SSVEP responses show peaks around 10 Hz and 20 Hz in most cases when using mid-range stimulation frequencies. All of these findings agree with the results of this study. Generally, the SSVEP can be influenced by the device and screen characteristics used (Zhu et al., 2010). This fact also explains the presence of a peak in the range of 15–20 Hz instead of strictly at 15 Hz (the target frequency), or in the range of 15–18 Hz instead of strictly at 18 Hz (the target frequency). For instance, when selecting 15 Hz for the flickering square, this value may vary from one device to another based on the screen characteristics. However, all these factors do not affect the clarity of the subject’s choice.

Moreover, the classification results showed high accuracy for all three classifiers (DT, SVM, KNN) with an accuracy percentage exceeding 75% using the feature sets of PSD, CCA, and combined PSD + CCA. Generally, the highest accuracy is achieved by the SVM classifier, reaching 93.75% ± 1.5% at 15 Hz and 87.5% ± 1.2% at 18 Hz with the combined feature set (PSD + CCA). Previous studies, such as (Wang et al., 2014), suggest that SSVEP classification could be enhanced by combining PSD and CCA, as the classification results from these two methods are somewhat correlated. This study confirms that combining PSD and CCA can indeed yield higher accuracy in classification. The superior classification capability of SVM classifier in this study stems from its effectiveness in managing high-dimensional feature spaces efficiently, a renowned characteristic that contributes to its exceptional performance when integrating multiple feature sets (Cervantes et al., 2020).

Generally, when comparing this novel method to conventional assessment methods, this innovative method offers unparalleled inclusivity, accommodating individuals worldwide, including those with disabilities that restrict movement or speech. Notably, individuals with locked-in syndrome, who may be fully paralyzed except for eye movement, find this method exceptionally well-suited, as it requires only the focus on a flickering square after identifying a target number. Furthermore, this method stands out for its simplicity, requiring no extensive training or detailed explanation, thereby saving valuable time. Its objective nature contributes to more reliable results compared to traditional tests.

When juxtaposed with previous research endeavors, this method distinguishes itself by its efficiency and user-friendliness. By significantly reducing the time and steps required for completion, it ensures the comfort of individuals undergoing the test. The straightforward approach is easily understandable across diverse populations, in contrast to the intricate techniques such as sweep SSVEP proposed in earlier studies (Zheng et al., 2021). These complex methods often necessitate prolonged experiment durations and data analysis, impeding their integration into routine clinical assessments. For instance, studies like (Norton et al., 2021) that delve into SSVEP experiments involving metamers identification tend to demand additional training or detailed explanations before testing, prolonging the overall testing process. In stark contrast, the method presented in this study requires no prior training or intense concentration, or fully mentally sound people, simply necessitating focus on the flickering square post-question posed by the tester. With a concise time frame not exceeding 30 min and a user-friendly approach that minimizes disturbances, this novel method is poised for seamless integration into clinical practices.

Moreover, by addressing the limitations associated with prior studies, such as Bieber et al. (1997) and Thomas et al. (2017), particularly in terms of demographic diversity encompassing age and gender, this method showcases its versatility by being successfully applied to individuals of varying demographics. Its effectiveness across both male and female subjects spanning different age ranges exemplifies its potential for widespread adoption in clinical settings. Table 4 presents a comparison of our study with recent literature on EEG-based methods for diagnosing or assessing color vision deficiency (CVD).

**TABLE 4:** Comparison of recent studies investigating EEG-based methods for diagnosing or assessing color vision deficiency (CVD).

Study	Year	Subjects	Objective	Key findings
Wicaksono et al. (2020)	2020	25 total (21 normal, 4 partial color-blind)	Investigate EEG response to Ishihara test stimulus in CVD vs normal vision using ERP	Significant differences found in ERP components (300–400 m) between CVD and normal individuals, primarily in parietal and occipital regions, suggesting impaired numerical processing in CVD participants
Zheng et al. (2021)	2020	26 total (15 CVD, 11 healthy)	Quantitative diagnosis of CVD using SSVEP and correlation with FM 100-hue test	SSVEP-based color vision severity index (ICVD) successfully classified between normal, anomalous trichromats, and dichromats; demonstrated strong correlation with traditional FM 100-hue test results
Ekhlasi et al. (2021)	2021	20 total (10 CVD, 10 healthy)	Analyze EEG signals during Ishihara’s test for CVD vs healthy individuals	Significant differences in frequency bands (Delta, Theta, Beta1, Beta2) between CVD and healthy groups in right temporoparietal regions (P4, T6); KNN classifier achieved 85.2% accuracy in distinguishing between groups
Norton et al. (2021)	2021	19 total (16 healthy, 3 CVD)	Develop a BCI-based method to assess color vision without active participation	SSVEP-based method to identify metamers allows for non-participatory assessment; demonstrated capability to distinguish between color-vision deficits and normal vision
Habibzadeh et al. (2022)	2022	10 healthy	Improve efficiency of BCI-based color vision assessment using metaID + algorithm	metaID + algorithm significantly reduced data requirements by 61.3% while achieving comparable accuracy to prior methods; enhanced SSVEP-based color vision assessment
Atkins et al. (2023)	2023	3 healthy	Optimize SSVEP stimulation frequency for BCI-based color vision assessment	Found 16 Hz to be the optimal stimulation frequency for differentiating CVD; results indicate potential for separate assessment of hue and illuminance effects on SSVEP responses
Current Study	2024	16 total (5 CVD, 11 healthy)	Diagnose CVD vs healthy using EEG	Achieved high accuracy using PSD and CCA features; SVM classifier showed best performance in distinguishing CVD from healthy controls

## 6 Limitations

Several limitations are associated with the current study that may impact the generalizability of the results. Firstly, the study included a small sample size with only 16 participants, five of whom are diagnosed with CVD. Although the diagnosis was successful for all CVD subjects, the consistency of outcomes and findings in this research should be further validated with a larger sample. Additionally, the limited number of participants increases the risk of overfitting, which could reduce the model’s performance on broader datasets. Secondly, the class imbalance favoring non-CVD participants poses challenges for machine learning algorithms, as these models may exhibit bias towards the majority class. To address this limitation, we used the SMOTE algorithm to balance the classes; however, future studies could explore alternative methods or employ specialized algorithms designed to handle imbalanced datasets. Lastly, the demographic constraints, with all CVD participants being male, limit the diversity of the sample and may affect generalizability across different populations. For future work, investigating CVD diagnosis with a large and gender-balanced sample would provide valuable insights into gender-specific patterns. These improvements aim to enhance the reliability and applicability of EEG-based CVD diagnostics.

## 7 Conclusion

In conclusion, the utilization of EEG signals and machine learning algorithms for the non-invasive classification and diagnosis of CVDs represents a significant advancement in the field of visual impairment assessment. This study has successfully showcased the effectiveness of employing SSVEP as a reliable method for diagnosing CVDs, particularly in individuals facing challenges with direct communication or behavioral responses. The findings of this research underscore the potential of SSVEP-based diagnostics to provide accurate and objective assessments of CVDs, circumventing the limitations associated with traditional diagnostic tests.

Moving forward, future investigations in this domain could focus on expanding the scope of color vision deficiencies considered, refining standardized operating protocols for EEG-based CVD diagnosis, validating the reliability of these methods across varied populations, and exploring the practical applications of such diagnostic techniques in clinical settings. Additionally, future studies might explore the integration of auditory or tactile BCIs to assist individuals with advanced locked-in syndrome who may have limited visual functionality. By continuing to advance research in this area, we can enhance the accessibility, accuracy, and inclusivity of CVD assessments, ultimately improving the quality of care and support provided to individuals with visual impairments.

## Data Availability

The raw data supporting the conclusions of this article will be made available by the authors, without undue reservation.

## References

[B1] Alamoudi N. B. AlShammari R. Z. AlOmar R. S. AlShamlan N. A. Alqahtani A. A. AlAmer N. A. (2021). Prevalence of color vision deficiency in medical students at a Saudi University. J. Fam. Community Med. 28 (3), 196–201. 10.4103/jfcm.jfcm_235_21 PMC8496699 34703380

[B2] Albahri A. S. Al-qaysi Z. T. Alzubaidi L. Alnoor A. Albahri O. S. Alamoodi A. H. (2023). A systematic review of using deep learning Technology in the steady-state visually evoked potential-based brain-computer interface applications: current trends and future trust methodology. Int. J. Telemedicine Appl. 2023, 1–24. 10.1155/2023/7741735 PMC10164869 37168809

[B3] AlEssa G. N. Alzahrani S. I. (2024). EEG-based methods for diagnosing color vision deficiency: a comprehensive review. Appl. Sci. 14 (17), 7579. 10.3390/app14177579

[B4] Atkins A. E. Habibzadeh H. Vaughan T. M. Norton J. J. (2023). “Optimizing stimulation frequency for BCI-based color vision assessment: preliminary results,” in Proc. 2023 11th int. IEEE/EMBS conf. Neural engineering (NER), 1–4. 10.1109/NER52421.2023.10123803

[B5] Bieber M. L. Knoblauch K. Werner J. S. (1997). “Detecting colour vision deficiency in 4- and 8-week-old human infants,” in Documenta ophthalmologica proceedings series colour vision deficiencies XIII: proceedings of the thirteenth symposium of the international research group on colour vision deficiencies, held in pau, France july 27–30, 1995. Editors Cavonius C. R. (Dordrecht: Springer Netherlands), 277–282. 10.1007/978-94-011-5408-6_29

[B6] Birch J. (1997). Efficiency of the Ishihara test for identifying red-green colour deficiency. Ophthalmic Physiological Opt. 17 (5), 403–408. 10.1016/S0275-5408(97)00022-7 9390366

[B7] Boateng E. Y. Otoo J. Abaye D. A. (2020). Basic tenets of classification algorithms K-Nearest-Neighbor, support vector machine, random forest and neural network: a review. J. Data Analysis Inf. Process. 8 (4), 341–357. 10.4236/jdaip.2020.84020

[B8] Britannica (2024). Information theory - entropy, data compression, communication | britannica. Available at: https://www.britannica.com/science/information-theory/Entropy (Accessed August 31, 2024).

[B9] Cao T. Wan F. Mak P. U. Mak P.-I. Vai M. I. Hu Y. (2012). “Flashing color on the performance of SSVEP-based brain-computer interfaces,” in 2012 annual international conference of the (IEEE Engineering in Medicine and Biology Society), 1819–1822. 10.1109/EMBC.2012.6346304 23366265

[B10] Celesia G. G. (2005). “Chapter 13 Color vision deficiencies,” in , Handbook of clinical neurophysiology. Editors Celesia G. G. (Elsevier), 251–269. 10.1016/S1567-4231(09)70210-2

[B11] Cervantes J. Garcia-Lamont F. Rodríguez-Mazahua L. Lopez A. (2020). A comprehensive survey on support vector machine classification: applications, challenges and trends. Neurocomputing 408, 189–215. 10.1016/j.neucom.2019.10.118

[B12] Chan X. B. V. Goh S. M. S. Tan N. C. (2014). Subjects with colour vision deficiency in the community: what do primary care physicians need to know? Asia Pac. Fam. Med. 13 (1), 10. 10.1186/s12930-014-0010-3

[B13] Chawla N. V. Bowyer K. W. Hall L. O. Kegelmeyer W. P. (2002). SMOTE: synthetic minority over-sampling technique. J. Artif. Intell. Res. 16, 321–357. 10.1613/jair.953

[B14] Chu L. Fernandez-Vargas J. Kita K. Yu W. (2017). “Influence of stimulus color on steady state visual evoked potentials,” in Presented at the advances in intelligent systems and computing, 499–509. 10.1007/978-3-319-48036-7_36

[B15] Colorlite (2024). Ishihara test. Colorlite | color blind glasses | color blind test. Available at: https://www.colorlitelens.com/ishihara-test.html (Accessed May 20, 2024).

[B16] Conway B. R. (2009). Color vision, cones, and color-coding in the cortex. Neuroscientist 15 (3), 274–290. 10.1177/1073858408331369 19436076

[B17] Covington B. P. Al Khalili Y. (2024). “Neuroanatomy, nucleus lateral geniculate,” in StatPearls (Treasure Island (FL): StatPearls Publishing). Available at: http://www.ncbi.nlm.nih.gov/books/NBK541137/. 31082181

[B18] Davidoff C. Neitz M. Neitz J. (2016). Genetic testing as a new standard for clinical diagnosis of color vision deficiencies. Transl. Vis. Sci. Technol. 5 (5), 2. 10.1167/tvst.5.5.2 PMC5017313 27622081

[B19] Decision Tree (2024). Decision tree. Available at: https://www.saedsayad.com/decision_tree.htm (Accessed July 23, 2024).

[B20] Dohvoma V. A. Ebana Mvogo S. R. Kagmeni G. Emini N. R. Epee E. Mvogo C. E. (2018). Color vision deficiency among biomedical students: a cross-sectional study. Clin. Ophthalmol. 12, 1121–1124. 10.2147/OPTH.S160110 29950807 PMC6016265

[B21] Du Y. Zhao X. (2022). Visual stimulus color effect on SSVEP-BCI in augmented reality. Biomed. Signal Process. Control 78, 103906. 10.1016/j.bspc.2022.103906

[B22] Duart X. Quiles E. Suay F. Chio N. García E. Morant F. (2020). Evaluating the effect of stimuli color and frequency on SSVEP. Sensors (Basel) 21 (1), 117. 10.3390/s21010117 33375441 PMC7796402

[B23] Ekhlasi A. Ahmadi H. Molavi A. Nia M. Motie Nasrabadi A. (2021). EEG signal analysis during Ishihara’s test in subjects with normal vision and color vision deficiency. Biomed. Phys. and Eng. Express 7, 025008. 10.1088/2057-1976/abdbbc 33445166

[B24] emhj (2021). Prevalence and predictors of colour vision defects among Egyptian university students. Cairo, Egypt: World Health Organization - Regional Office for the Eastern Mediterranean. Available at: http://www.emro.who.int/emhj-volume-27-2021/volume-27-issue-4/prevalence-and-predictors-of-colour-vision-defects-among-egyptian-university-students.html. 10.26719/emhj.20.128 33955536

[B25] Fakorede S. T. Akpan L. G. Adekoya K. O. Oboh B. (2022). Prevalence and population genetic data of colour vision deficiency among students from selected tertiary institutions in Lagos State, Nigeria. Egypt. J. Med. Hum. Genet. 23 (1), 73. 10.1186/s43042-022-00287-9

[B26] Fanlo Zarazaga A. Gutiérrez Vásquez J. Pueyo Royo V. (2019). Review of the main colour vision clinical assessment tests. Arch. Soc. Española Oftalmol. (English Ed.) 94 (1), 25–32. 10.1016/j.oftale.2018.08.010 30361001

[B27] Fareed M. Anwar M. A. Afzal M. (2015). Prevalence and gene frequency of color vision impairments among children of six populations from North Indian region. Genes and Dis. 2 (2), 211–218. 10.1016/j.gendis.2015.02.006 PMC6150100 30258865

[B28] Göksel Duru D. Alobaidi M. (2021). Classification of brain electrophysiological changes in response to colour stimuli. Phys. Eng. Sci. Med. 44 (3), 727–743. 10.1007/s13246-021-01021-2 34269986

[B29] Gordon N. (1998). Colour blindness. Public Health 112 (2), 81–84. 10.1038/sj.ph.1900446 9581449

[B30] Gupta A. Laxmi G. Nittala M. G. Raman R. (2011). Structural and functional correlates in color vision deficiency. Eye 25 (7), 909–917. 10.1038/eye.2011.87 21494280 PMC3178173

[B31] Habibzadeh H. Long K. J. Atkins A. E. Zois D. S. Norton J. J. (2022). “Improving BCI-based color vision assessment using Gaussian process regression,” in Proc. ICASSP 2022 - 2022 IEEE international conference on acoustics, speech and signal processing, 1306–1310. 10.1109/ICASSP43922.2022.9747015

[B32] Hakvoort G. (2010). Comparison of PSDA and CCA detection methods in a SSVEP-based BCI-system, 11.

[B33] He F. Abu Bakar A. (2023). “Optimization of stimulus color for SSVEP-based brain-computer interfaces in mixed reality,” in Human brain and artificial intelligence. Editor Ying X. (Singapore: Springer Nature), 183–191. 10.1007/978-981-19-8222-4_16

[B34] Hendrickson A. Bumsted-O”Brien K. Natoli R. Ramamurthy V. Possin D. Provis J. (2008). Rod photoreceptor differentiation in fetal and infant human retina. Exp. Eye Res. 87 (5), 415–426. 10.1016/j.exer.2008.07.016 18778702 PMC4122835

[B35] Hidden plates (2024). Hidden plates. Available at: https://www.color-blind-test.com/color-blind-tests/ishihara-test/hidden-plates.html (Accessed May 20, 2024).

[B36] Károly L. (2024). “Farnsworth D-15 color blind test,” colorlite | color blind glasses | color blind test. Available at: https://www.colorlitelens.com/d15-color-blind-test-more.

[B37] Knyazev G. G. Slobodskoj-Plusnin J. Y. Bocharov A. V. Pylkova L. V. (2011). The default mode network and EEG alpha oscillations: an independent component analysis. Brain Res. 1402, 67–79. 10.1016/j.brainres.2011.05.052 21683942

[B38] Kuś R. Duszyk A. Milanowski P. Łabęcki M. Bierzyńska M. Radzikowska Z. (2013). On the quantification of SSVEP frequency responses in human EEG in realistic BCI conditions. PLoS One 8 (10), e77536. 10.1371/journal.pone.0077536 24204862 PMC3799619

[B39] Ma P. Dong C. Lin R. Ma S. Jia T. Chen X. (2022). A classification algorithm of an SSVEP brain-Computer interface based on CCA fusion wavelet coefficients. J. Neurosci. Methods 371, 109502. 10.1016/j.jneumeth.2022.109502 35151665

[B40] Male S. R. B R S. Gandhi R. Theagarayan B. (2023). Global prevalence of color vision deficiency: a systematic review and meta-analysis. Investigative Ophthalmol. and Vis. Sci. 64 (8), 1508.

[B41] Miyahara E. (2009). Chromaticity co-ordinates of Ishihara plates reveal that hidden digit plates can be read by S-cones. Clin. Exp. Optometry 92 (5), 434–439. 10.1111/j.1444-0938.2009.00396.x 19558531

[B42] Nguyen K. T. Liang W.-K. Muggleton N. G. Huang N. E. Juan C.-H. (2019). Human visual steady-state responses to amplitude-modulated flicker: latency measurement. J. Vis. 19 (14), 14. 10.1167/19.14.14 31845974

[B43] Norcia A. M. Appelbaum L. G. Ales J. M. Cottereau B. R. Rossion B. (2015). The steady-state visual evoked potential in vision research: a review. J. Vis. 15 (6), 4. 10.1167/15.6.4 PMC4581566 26024451

[B44] Norton J. J. S. DiRisio G. F. Carp J. S. Norton A. E. Kochan N. S. Wolpaw J. R. (2021). Brain-computer interface-based assessment of color vision. J. Neural Eng. 18, 066024. 10.1088/1741-2552/ac3264 PMC9094738 34678801

[B45] OpenBCI (2023). OpenBCI | home. Available at: https://openbci.com/.

[B46] Pandey N. Chandrakar A. K. Garg M. L. (2015). Tests for color vision deficiency: is it time to revise the standards? Indian J. Ophthalmol. 63 (9), 752–753. 10.4103/0301-4738.170975 26632139 PMC4705720

[B47] Park E. Cho M. Ki C. S. (2009). Correct use of repeated measures analysis of variance. Korean J. Laboratory Med. 29 (1), 1–9. 10.3343/kjlm.2009.29.1.1 19262072

[B48] Regan D. (1975). Recent advances in electrical recording from the human brain. Nature 253 (5491), 401–407. 10.1038/253401a0 1089209

[B49] Roy S. Banerjee A. Roy C. Nag S. Sanyal S. Sengupta R. (2021). Brain response to color stimuli: an EEG study with nonlinear approach. Cogn. Neurodyn 15 (6), 1023–1053. 10.1007/s11571-021-09692-z 34790269 PMC8572309

[B50] Ryans Computers (2024). What is the meaning of response time (ms) 1ms (gray to gray)? | ryans computers. Available at: https://www.ryans.com/glossary/response-time-ms-1ms-gray-to-gray (Accessed July 01, 2024).

[B51] Salvia J. Ysseldyke J. (1971). An analysis of the reliability and validity of the Ishihara color plates with mentally retarded males. Percept. Mot. Ski. 33 (1), 243–246. 10.2466/pms.1971.33.1.243 5315329

[B52] Scarpino O. Guidi M. Bolcioni G. (1990). Topographic EEG analysis. Methods for graphic representation and clinical applications. Acta Neurol. (Napoli) 12 (5), 410–426. 2082724

[B53] ScienceDirect Topics (2024). Visual pathway - an overview | ScienceDirect Topics. Available at: https://www.sciencedirect.com/topics/neuroscience/visual-pathway.

[B54] Shah A. Hussain R. Fareed M. Afzal M. (2013). Prevalence of red-green color vision defects among muslim males and females of Manipur, India. Iran. J. Public Health 42 (1), 16–24. 23515069 PMC3595632

[B55] Souza-Couto D. Bretas R. Aversi-Ferreira T. A. (2023). Neuropsychology of the parietal lobe: luria’s and contemporary conceptions. Front. Neurosci. 17, 1226226. 10.3389/fnins.2023.1226226 37928730 PMC10623013

[B56] Srinivasan R. Bibi F. A. Nunez P. L. (2006). Steady-state visual evoked potentials: distributed local sources and wave-like dynamics are sensitive to flicker frequency. Brain Topogr. 18 (3), 167–187. 10.1007/s10548-006-0267-4 16544207 PMC1995016

[B57] Storr W. (2024). “Optocoupler tutorial and optocoupler application,” in Basic electronics tutorials. Available at: https://www.electronics-tutorials.ws/blog/optocoupler.html (Accessed: May 01, 2024).

[B58] Teixeira A. R. Gomes A. (2023). “Analysis of visual patterns through the EEG signal: color study,” in Augmented cognition: 17th international conference, AC 2023, held as part of the 25th HCI international conference, HCII 2023, Copenhagen, Denmark, july 23–28, 2023, proceedings (Berlin, Heidelberg: Springer-Verlag), 46–53. 10.1007/978-3-031-35017-7_4

[B59] Thomas B. Rajendran R. Koganti Y. Maheswari V. U. (2017). Portable embedded device to analyse the effect of color blindness on EEG. Available at: https://ieeexplore.ieee.org/document/8067946.

[B60] Thomas B. Umamaheswari V. (2016). EEG based color impairment detection. Int. J. Recent Innovation Trends Comput. Commun. 4 (3), 540–542. 10.17762/ijritcc.v4i3.1934

[B61] Thoreson W. B. Dacey D. M. (2019). Diverse cell types, circuits, and mechanisms for color vision in the vertebrate retina. Physiol. Rev. 99 (3), 1527–1573. 10.1152/physrev.00027.2018 31140374 PMC6689740

[B62] Uddin S. Khan A. Hossain M. E. Moni M. A. (2019). Comparing different supervised machine learning algorithms for disease prediction. BMC Med. Inf. Decis. Mak. 19, 281. 10.1186/s12911-019-1004-8 PMC6925840 31864346

[B63] Vinekar A. Mangalesh S. Jayadev C. Maldonado R. S. Bauer N. Toth C. A. (2015). Retinal imaging of infants on spectral domain optical coherence Tomography. BioMed Res. Int. 2015 (1), 1–12. 10.1155/2015/782420 PMC4506845 26221606

[B64] Wang R. Wu W. Iramina K. Ge S. (2014). “The combination of CCA and PSDA detection methods in a SSVEP-BCI system,” in Proceeding of the 11th world congress on intelligent control and automation, 2424–2427. 10.1109/WCICA.2014.7053101

[B65] Welch’s Method (2024). Welch’s method. Available at: https://ccrma.stanford.edu/∼jos/sasp/Welch_s_Method.html.

[B66] Werner A. (2014). Spatial and temporal aspects of chromatic adaptation and their functional significance for colour constancy. Vis. Res. 104, 80–89. 10.1016/j.visres.2014.10.005 25449338

[B67] Wicaksono A. Mengko T. Iramina K. (2020). “Investigation of EEG signal response using event-related potential (ERP) towards Ishihara pseudo-isochromatic visual stimulus,” in Proc. IEEE signal processing in medicine and biology symposium (SPMB), 1–5. 10.1109/SPMB50085.2020.9353640

[B68] Woldeamanuel G. G. Geta T. G. (2018). Prevalence of color vision deficiency among school children in Wolkite, Southern Ethiopia. BMC Res. Notes 11 (1), 838. 10.1186/s13104-018-3943-z 30486898 PMC6263558

[B69] Xie S. Liu C. Obermayer K. Zhu F. Wang L. Xie X. (2016). Stimulator selection in SSVEP-based spatial selective attention study. Comput. Intell. Neurosci. 2016 (1), 1–9. 10.1155/2016/6410718 PMC5156793 28044073

[B70] Zhang X. Xu G. Xie J. Zhang X. (2017). Brain response to luminance-based and motion-based stimulation using inter-modulation frequencies. PLoS One 12 (11), e0188073. 10.1371/journal.pone.0188073 29141030 PMC5687765

[B71] Zheng X. Xu G. Wang Y. Du C. Liang R. Zhang K. (2021). Quantitative and objective diagnosis of color vision deficiencies based on steady-state visual evoked potentials. Int. Ophthalmol. 41 (2), 587–598. 10.1007/s10792-020-01613-z 33044670

[B72] Zhu D. Bieger J. Garcia Molina G. Aarts R. M. (2010). A survey of stimulation methods used in SSVEP-based BCIs. Comput. Intell. Neurosci. 2010 (1), 1–12. 10.1155/2010/702357 PMC2833411 20224799

